# A Novel Antigen Design Strategy to Isolate Single‐Domain Antibodies that Target Human Nav1.7 and Reduce Pain in Animal Models

**DOI:** 10.1002/advs.202405432

**Published:** 2024-08-29

**Authors:** Marzia Martina, Umberto Banderali, Alvaro Yogi, Mehdi Arbabi Ghahroudi, Hong Liu, Traian Sulea, Yves Durocher, Greg Hussack, Henk van Faassen, Balu Chakravarty, Qing Yan Liu, Umar Iqbal, Binbing Ling, Etienne Lessard, Joey Sheff, Anna Robotham, Debbie Callaghan, Maria Moreno, Tanya Comas, Dao Ly, Danica Stanimirovic

**Affiliations:** ^1^ Human Health Therapeutics Research Center National Research Council Canada 1200 Montreal Road, Building M54 Ottawa ON K1A 0R6 Canada; ^2^ Human Health Therapeutics Research Centre National Research Council Canada 100 Sussex Drive Ottawa ON K1N 5A2 Canada; ^3^ Human Health Therapeutics Research Centre National Research Council Canada 6100 Royalmount Avenue Montréal Quebec H4P 2R2 Canada

**Keywords:** automated patch‐clamp system, epitope, mouse OD1 model, pain, rat Hargreaves model, surface plasmon resonance, V_H_H

## Abstract

Genetic studies have identified the voltage‐gated sodium channel 1.7 (**Na**_**v**_ **1.7**) as pain target. Due to the ineffectiveness of small molecules and monoclonal antibodies as therapeutics for pain, single‐domain antibodies (**V**_**H**_ **Hs**) are developed against the human Na_v_1.7 (**hNa_v_1.7**) using a novel antigen presentation strategy. A 70 amino‐acid peptide from the hNa_v_1.7 protein is identified as a target antigen. A recombinant version of this peptide is grafted into the complementarity determining region 3 (**CDR3**) loop of an inert V_H_H in order to maintain the native 3D conformation of the peptide. This antigen is used to isolate one V_H_H able to i) bind hNa_v_1.7, ii) slow the deactivation of hNa_v_1.7, iii) reduce the ability of eliciting action potentials in nociceptors, and iv) reverse hyperalgesia in in vivo rat and mouse models. This V_H_H exhibits the potential to be developed as a therapeutic capable of suppressing pain. This novel antigen presentation strategy can be applied to develop biologics against other difficult targets such as ion channels, transporters and GPCRs.

## Graphical abstract

An innovative antigen presentation strategy is used to isolate a single domain antibody (**V_H_H**) against the human voltage‐gated sodium channel 1.7 (**hNa**_**v**_ **1.7**) channel, from a phage‐displayed naïve V_H_H library generated from llama, alpaca and camel. This V_H_H is able to modulate hNa_v_1.7 currents, reduce the ability of eliciting action potentials in nociceptors and reverse hyperalgesia in in vivo rodent pain models.

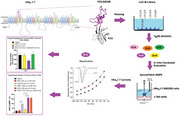

## 1 Introduction

Sodium channels (**Na_v_**) are involved in the control of many fundamental physiological processes in various tissues of the human body. In excitable cells, Na_v_s are essential for the initiation and propagation of electrical signals. The Na_v_ family has nine types of pore‐forming *α* subunits, which in mammals are numbered from Na_v_1.1 to 1.9 and are encoded by the genes SCN1A, SCN2A, SCN3A, SCN4A, SCN5A, SCN8A, SCN9A, SCN10A, and SCN11A, respectively.^[^ ^1^ ^]^ These isoforms share a common overall structural motif, with each Na_v_ type exhibiting a different function and expression profile. Na_v_1.1, Na_v_1.2, Na_v_1.3, and Na_v_1.6 are mainly expressed in the **CNS** (central nervous system).^[^ ^2^, ^3^, ^4^, ^5^ ^]^ Na_v_1.7, Na_v_1.8, and Na_v_1.9 are present in the **PNS** (peripheral nervous system), with these channels further accumulating in the region of peripheral nerve injury, and consequently may be important in propagating chronic and neuropathic pain.^[^ ^6^, ^7^ ^]^ Na_v_1.4 is the muscle sodium channel and Na_v_1.5 is the predominant cardiac myocyte channel.

The Na_v_1.7 channel is expressed in nociceptive neurons (**DRG**, dorsal root ganglion,) of the PNS.^[^ ^8^ ^]^ Nociceptive stimuli (i.e., injury or inflammation) are initiated at peripheral receptors, and then transduced at peripheral termini by Na_v_ dependent action potentials. Na_v_1.7 channels are also present in the central axonal projections of DRG neurons and their presynaptic terminals within the dorsal horn of the spinal cord, facilitating impulse invasion or evoked release of neurotransmitters^[^ ^9^ ^]^ Homology modelling based on crystal structures of ion channels suggests an atomic‐level structural basis for the altered gating of mutant Na_v_1.7 that causes pain.^[^ ^5^ ^]^ Genetic, structural and functional studies have shown that Na_v_1.7 regulates sensory neuron excitability – a major contributor to several sensory modalities – and have established the contribution of Na_v_1.7 to human pain disorders.^[^ ^5^ ^]^ In particular, genetic studies show that mutations in SCN9A gene produce familial pain disorders.^[^ ^10^ ^]^ These mutations can be described as gain of function mutations (increase of Na_v_1.7 activity) which cause **PEPD** (paroxysmal extreme pain disorder) and **EM** (erythromelalgia), or loss of function mutations (reduction of Na_v_1.7 activity) which cause **CIP** (congenital insensitivity to pain).^[^ ^11^ ^]^ Importantly, people totally lacking Na_v_1.7 have minimal cognitive, cardiac, motor and sensory deficits, supporting Na_v_1.7 as a valid and indeed attractive target for development of drugs against pain. Na_v_1.7 activity is also increased in preclinical rodent models of chronic neuropathic pain.^[^ ^12^, ^13^, ^14^ ^]^

While many pain forms are well managed with current medications, some forms of chronic pain, including neuropathic pain and cancer‐induced pain, are often insensitive to these medications.^[^ ^15^ ^]^ In addition, several current medications for managing chronic pain, notably opioids, can induce addiction and abuse, and are a serious global problem affecting the health, social, and economic welfare of all societies.^[^ ^16^ ^]^

**Figure 1:**
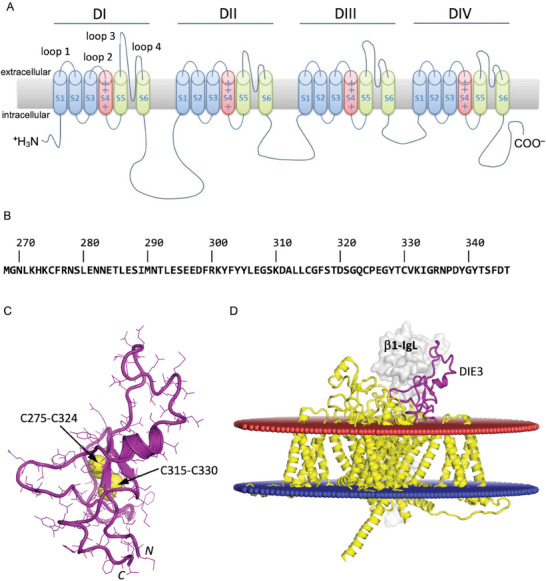
Location, sequence and structure of selected epitope on human Na_v_1.7 channel. A) Schematic representation of channel structure. B) Amino‐acid sequence of extracellular Loop 3 of domain DI (DIE3) with selected epitope in bold (Uniprot ID Q15858). C) Modeled 3D structure of DIE3 loop including two disulfide bonds (labeled). D) 3D‐location of DIE3 loop (purple) relative to cell membrane (red/blue planes) shown on the cryo‐EM structure (PDB ID 5XSY) of the homologous electric eel Na_v_1.4 channel alpha subunit (yellow) complexed with *β*1 subunit (gray surface).

A novel type of pain therapeutic with the ability of inhibiting Na_v_1.7 with high specificity could be a potential solution to the problem. However, the development of such a therapeutic represents a challenge due to the high degree of amino‐acid sequence homology among the different Na_v_ subtypes.^[^ ^1^ ^]^ Na_v_s are comprised of one *α* subunit and two auxiliary *β* subunits. The *α* subunit is composed of four domains (DI to DIV) forming a pseudo‐tetrameric structure. Each domain possesses six transmembrane spanning helices (S1–S6) and a pore region between S5 and S6 of each domain.^[^ ^17^ ^]^ Each domain has a **VSD** (Voltage Sensing Domain) formed by S1–S4, which regulates the opening of the channel during depolarization of the membrane. The *α* subunit also contains the inactivation gate formed by the intracellular loop connecting DIII and DIV (**Figure** **1**A). The auxiliary *β* subunits have multiple functions including the trafficking of the protein complex to the plasma membrane. Six different *β* subunits have been identified: *β*1, *β*2, *β*3, *β*4, and *β*1A and *β*1B that are splice variants of *β*1. All *β* subunits affect the channel properties of Na_v_1.7, although *β*1 and *β*2 are more commonly co‐expressed with the Na_v_1.7 *α* subunit for biophysical characterization.^[^ ^18^, ^19^ ^]^ In addition, Na_v_s exhibit different dynamic states (closed, open, or inactivated).

In the last decade, highly selective small molecules and peptide toxins targeting hNa_v_1.7 have been developed as pain therapeutics.^[^ ^20^ ^]^ However, these compounds displayed very limited analgesic effect in animal pain models and were not further pursued as therapeutics.^[^ ^21^ ^]^ Monoclonal antibodies (**mAbs**) against hNa_v_1.7 were also developed. However, these mAbs are usually binders (i.e., SVmab) lacking functional efficacy.

**V_H_Hs** (single‐domain antibodies, also known as nanobodies) are 12–15 kDa proteins derived from the variable domains of heavy‐chain‐only antibodies. V_H_Hs are well suited for various applications due to possessing favorable characteristics such as small size, ease of genetic manipulation, high affinity and solubility, overall stability, resistance to harsh conditions (e.g., low pH, high temperature), and low immunogenicity.^[^ ^22^ ^]^ Most importantly, V_H_Hs can penetrate cavities, thereby recognizing hidden epitopes normally inaccessible to conventional antibodies. Due to these properties, V_H_Hs can be produced to target specific subunits or epitopes or the state of a channel with a high degree of specificity. We therefore decided to develop V_H_Hs against hNa_v_1.7 channel.

A large body of evidence regarding the sequences containing the lowest homology between the subtypes of human Na_v_ channels, the regions containing mutations causing CIP and the interaction regions for pore blockers (i.e., small molecules and natural toxins) are now available to guide the development of new inhibitors.^[^ ^11^, ^23^ ^]^ Structural information associated with multiple function‐related conformations of Na_v_ channels is also indispensable for understanding the underlying molecular mechanism of gating.^[^ ^24^ ^]^ We used this knowledge to design a novel antigen presentation strategy to isolate V_H_Hs targeting Na_v_1.7 from a phage‐displayed naïve V_H_H library generated from llama, alpaca and camel (**LAC‐M library**). We identified one V_H_H that slowed the deactivation kinetic of hNa_v_1.7 currents, reduced the ability of eliciting action potentials in nociceptors and reduced hyperalgesia in in vivo animal models. In addition, this V_H_H could be potentially used to developed a therapeutic capable of suppressing pain.

## 2 Results

### 2.1 Engineering of Extracellular Na_v_1.7 Loop for Raising Highly Specific Antibodies

We examined mutations in the gene coding for the *α* subunit of hNa_v_1.7 (SCN9A) associated with CIP to identify an extracellular amino‐acid sequence that could be targeted by V_H_Hs to modulate hNa_v_1.7 currents. Since the 3D structure of hNa_v_1.7 was not yet available at the beginning of this study, we examined the crystal structure of i) a bacterial sodium channel Na_v_Ab,^[^ ^17^, ^24^ ^]^ ii) the single‐particle cryo‐electron microscopy (**cryo‐EM**) structures of eukaryotic sodium channels Na_v_PaS from American cockroach,^[^ ^25^ ^]^ and iii) Na_v_1.4 from electric eel^[^ ^26^ ^]^ to infer the 3D structure of hNa_v_1.7. A region of 70 amino‐acid residues (Figure 1B) from the domain DI extracellular loop 3 (termed DIE3IR in this study) of the wild‐type hNa_v_1.7 was selected for several reasons. First, this loop is distinct among the family of human sodium channels, with the closest homologous extracellular loop belonging to human Na_v_1.2 (SCN2A) expressing only 60% protein sequence identity (see Results Section 2.6. DI‐D selectivity). Second, this region corresponds to the sequence that contains mutations linked to CIP (Y328X and R277X).^[^ ^27^ ^]^ Third, this extracellular segment is predicted to form a well‐folded globular domain stabilized by two disulphide bonds and to have its N‐ and C‐termini in close proximity (Figure 1C), based on its high homology to the recent cryo‐EM structures of eukaryotic sodium channels (Figure S1A, Supporting Information). Finally, this solvent accessible extracellular domain appears to be prone to interactions with immunoglobulin (**Ig**) folded structures, based on the cryo‐EM structure of eel Na_v_1.4 complexed with the Ig‐like *β*1 subunit (Figure 1D), which we found to be relevant for our immunization attempts. The 3D structural information gleaned from homology to eukaryotic channels available was later validated and remained consistent with the recent cryo‐EM structures of the hNa_v_1.7.^[^ ^19^, ^28^ ^]^

**Figure 2:**
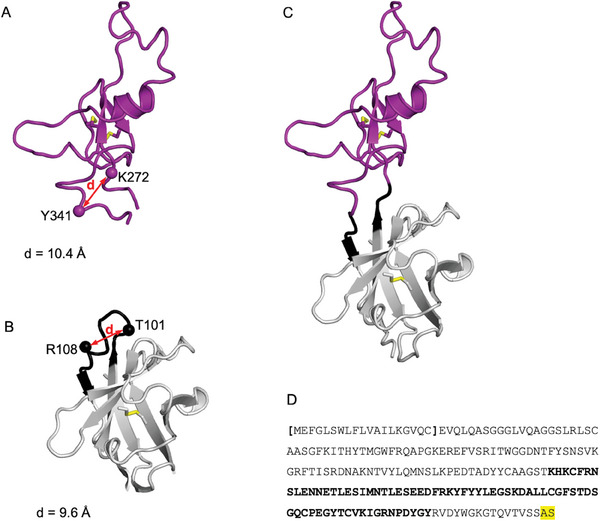
Immunogen design by DIE3 loop grafting into nanobody framework. A) 3D molecular model of human Na_v_1.7 DIE3 loop. The flexible termini located outside the indicated positions were removed for grafting. The distance between the new loop termini is indicated. B) 3D molecular model of the V_H_H FC5 nanobody with the hypervariable CDR3 loop highlighted in black. The inner region of the CDR3 loop between indicated positions was removed for grafting. The distance between the receiving anchors on the FC5 nanobody matches the distance between the selected termini of the DIE3IR loop graft. C) 3D molecular model of the designed immunogen including 70 amino‐acid residues of the DIE3IR (purple) grafted on the FC5 nanobody framework (gray). Seven amino‐acid residues from the original CDR3 of FC5 were retained (black), 3 on the N‐terminal side and 4 on the C‐terminal side of the grafted DIE3IR peptide. D) Amino‐acid sequence of the recombinant designed immunogen construct with the signal peptide in brackets and the DIE3 sequence highlighted in bold.

In order to further stabilize the native 3D structure of the soluble form of the DIE3IR loop in the absence of the rest of the hNa_v_1.7 channel (**Figure** **2**A), we grafted it inside the complementarity determining region 3 (**CDR3**) loop of the llama V_H_H FC5 (Figure 2B),^[^ ^29^ ^]^ thus producing the recombinant protein FC5‐DIE3IR (Figure 2C,D).

We hypothesized that the inner region of the hypervariable CDR3 loop of V_H_Hs would tolerate sequence changes without perturbing the overall folding of the V_H_H structure, thereby providing rigid anchors for the constraining N‐ and C‐termini of the grafted DIE3IR domain. We selected the FC5 as nanobody scaffold for its inherent tolerance to large sequence variations in the hypervariable CDR3 loop in addition to other biophysical properties including folding stability.^[^ ^30^ ^]^ In addition, structural models used for immunogen design as well as the recent 3D structures solved for hNa_v_1.7^[^ ^19^, ^28^ ^]^ indicated a certain degree of stabilizing intramolecular interactions within the selected loop for grafting, including two disulfide bonds and several short‐range packing interactions.

In the design of this recombinant protein construct (see Experimental Section), geometric measurements on 3D molecular models of DIE3IR loop domain and V_H_H FC5 guided the selection of appropriate grafting points on both molecules to be fused by distance matching (Figure 2A,B). Thus, 6 amino‐acid residues in the middle of the CDR3 of FC5 were removed (Figure 2B) and replaced by the 70‐amino‐acid DIE3 sequence which lacked the flexible, membrane‐proximal, ends of the entire DIE3IR loop (Figures 1B and 2A). Seven amino‐acid residues from the original CDR3 of FC5 were retained, 3 on the N‐terminal side and 4 on the C‐terminal side of the grafted DIE3IR peptide, in an attempt to maximize the accessibility of the grafted DIE3IR domain for antibody recognition and to minimize possible alterations of its native folding due to proximity to the FC5 structure (Figure 2C).

The recombinant protein FC5‐DIE3IR was then produced in a Chinese hamster ovary (**CHO**) cell line expressing a truncated EBNA1 protein (CHO‐3E7; Experimental Section; Figure S2A, Supporting Information).^[^ ^31^ ^]^ The SDS‐PAGE gel for FC5‐DIE3IR showed a smear (Figure S2A, Supporting Information). To verify whether this was due to glycosylation rather than protein truncation, we performed protein analysis using intact Mass Spectrometry (**MS**). The MS data shows that i) the native FC5‐DIE3IR is indeed glycosylated (about up to 50%), and that ii) FC5‐DIE3IR treated with PNGase F yields a homogeneous protein population (Figure S2B,C, Supporting Information). This glycosylation pattern observed by MS correlates with the heterogeneity/smears observed in the gel.

### 2.2 Selection of Functional V_H_Hs

We used the recombinant protein FC5‐DIE3IR to isolate V_H_Hs specific to DIE3 loop via panning of a naïve llama, alpaca, camel library (LAC‐M library; Experimental Section).^[^ ^22^ ^]^ The library was constructed from a pool of 30 different animal repertoires. We screened 76 colonies using polymerase chain reaction (**PCR**). By phage‐ELISA, 6 positive unique clones (DI‐A, DI‐B, DI‐C, DI‐D, DI‐E, DI‐H) with various degrees of sequence repetitions were identified. Five of the 6 V_H_Hs (Table S1, Supporting Information) were expressed in *E. coli* and purified by immobilized metal affinity chromatography (**IMAC**), after being gene‐synthesized and cloned by GenScript (Piscataway, NJ, USA). We were not able to produce any stable V_H_H DI‐E protein.

To verify the effect of the V_H_Hs on the hNa_v_1.7 currents, we generated **hNa_v_1.7‐HEK293 cells**(HEK293 cells over‐expressing hNa_v_1.7; Experimental Section; Figure S3, Supporting Information). The V_H_Hs were tested at the concentration of 2 µm on the Na^+^ currents evoked from hNa_v_1.7‐HEK293 cells in manual patch‐clamp experiments. This concentration is in the range of the equilibrium dissociation constants (**K_D_**) for epitope binding of V_H_Hs isolated from naïve libraries (see below).

**Figure 3:**
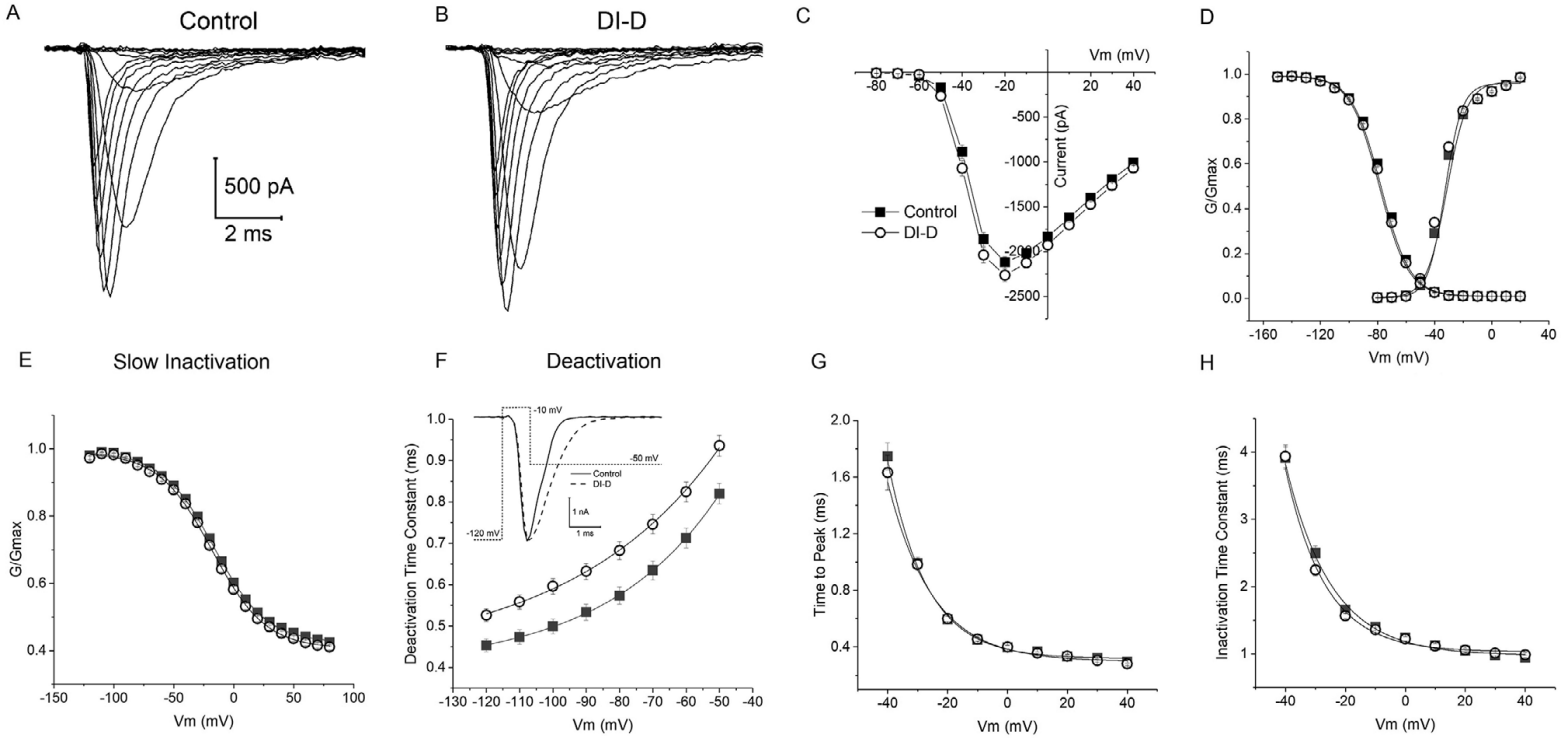
Effect of the application of V_H_H DI‐D on the kinetics of the Na_v_1.7 currents recorded using SyncroPatch 384PE in HEK293 cells overexpressing hNa_v_1.7 channels. Families of Na_v_1.7 currents recorded in voltage‐clamp using SyncroPatch 384PE in control A) and in presence of 2 µm V_H_H DI‐D B). C) Current–voltage (I–V) relationships showing peak current amplitude in control (full square; n = 278) and in the presence of V_H_H DI‐D (empty circles; n = 268). D) Activation and fast inactivation traces. E) Steady state slow inactivation. F) Voltage dependence of the deactivation currents decay. The inset displays an example of deactivation tail currents with superimposed voltage‐clamp protocol. G) Voltage dependence of time to peak. H) Voltage dependence of inactivation time constant. Statistics shown as mean ± SEM.

The hNa_v_1.7‐HEK293 cells were prepared from frozen vials and plated on coverslips. The coverslips were then mounted on a recording chamber and placed under an inverted microscope to perform the patch‐clamp experiments. Sodium currents recorded in hNa_v_1.7‐HEK293 cells at ‐20 mV displayed an average amplitude of 3555 ± 432 pA (n = 41; Figure S4, Supporting Information), significantly larger than the control HEK293 cells (385 ± 85 pA, n = 13; Figure S4, Supporting Information). The bath application of 2 µm of DI‐A, DI‐B, DI‐C, DI‐D and DI‐H did not change the amplitude of the Na^+^ currents in hNa_v_1.7‐HEK293 cells (**Figure** **3**, Figure S5, Supporting Information; Experimental Section). To assess the effect of the V_H_Hs on the kinetics and voltage dependence properties of the hNa_v_1.7 currents, we used the high‐throughput automated patch‐clamp system SyncroPatch 384 PE (see Experimental Section) and measured the current–voltage (**I–V**) relationship, the voltage‐dependence of activation, steady‐state fast inactivation, steady‐state slow inactivation, deactivation, time to peak and inactivation decay time in hNa_v_1.7‐HEK293 cells.

Of the 5 V_H_Hs tested, only V_H_H DI‐D had an effect on the kinetics of hNa_v_1.7. V_H_H DI‐D produced a significantly slower deactivation of hNa_v_1.7 currents (Figure 3; Figure S5, Supporting Information). As shown in Figure 3F, V_H_H DI‐D caused a significant increase in the time constant of deactivation current decay across all the potentials tested (from −120 to −50 mV). The time constant at −50 mV was 0.82 ± 0.03 ms (n = 202) in control and 0.94 ± 0.03 ms (n = 242) in the presence of V_H_H DI‐D (*p* = 0.004). V_H_H DI‐D had no effect on the I–V relationship, voltage‐dependence of activation, steady‐state fast inactivation, steady‐state slow inactivation, time to peak and inactivation decay time (Figure 3; Table S2, Supporting Information).

The slow inactivation has been reported to be characterized by a sequence of multiple slow‐inactivated states that depend on the duration of the depolarizing pre‐pulse. Consequently, the voltage dependence of channels availability also depends on the duration of the pre‐pulse.^[^ ^32^, ^33^ ^]^ The half slow inactivation voltage (**V1/2**) values we recorded (control, −16.4 ± 1.6, n = 278; V_H_H DI‐D, −17.9 ± 0.7, n = 268; Table S2, Supporting Information) were different from the V1/2 reported in literature (e.g.,^[^ ^34^ ^]^). This was due to an intrinsic limitation of the SycroPatch 384PE instrument we used. Indeed, we had to limit the duration of the slow‐inactivating pre‐pulse to 2 s and this yielded the V1/2 values given in Table S2 (Supporting Information). In order to verify and validate the slow inactivation process, we measured slow inactivation using manual patch‐clamp experiments with a pre‐pulse of 20 s. In these experiments (data not shown) the V1/2 was −60.6 ± 2.9 mV (n = 4) in control and −60.0 ± 1.4 mV (n = 5) in the presence of V_H_H DI‐D (2 µm), which is consistent with the data in literature.^[^ ^34^ ^]^

### 2.3 V_H_H DI‐D Binds DIE3IR

To demonstrate the specificity of V_H_H DI‐D binding to the DIE3IR loop peptide, ELISA and surface plasmon resonance (**SPR**) binding experiments were performed on the V_H_H DI‐D and the negative controls V_H_Hs FC5 and 2A3‐H4. We tested V_H_H DI‐D binding to various FC5‐DIE3IR constructs, where the grafted DIE3IR peptide corresponded to human, mouse or rat Na_v_1.7 channels. Verification of binding of V_H_H DI‐D to Na_v_1.7 from several species was necessary before proceeding with in vivo animal experiments. V_H_H DI‐D showed binding to rat, mouse and human FC5‐DIE3IR with similar affinities (Table S3 and Figure S6, Supporting Information), with equilibrium dissociation constant (***K*_D_**) values ranging from 1.56 to 1.72 µm for V_H_H DI‐D, which is typical for V_H_Hs isolated from naïve libraries. The observed *R*_max_ values ranged between 191 and 248 RUs, with theoretical *R*_max_ values of 290–309 RUs, indicating a high level of surface activity (61–84%) for all immobilized FC5‐DIE3IR species variants. In contrast, V_H_H DI‐D showed very weak binding to immobilized V_H_H FC5 and did not bind an irrelevant V_H_H 2A3‐H4.

Overall, these data confirm that V_H_H DI‐D binds the target loop DIE3IR from different species and does not bind to the V_H_H FC5 scaffold.

### 2.4 Target Engagement: Mouse OD1‐Induced Pain Model

To evaluate the engagement of the Na_v_1.7 channels by the V_H_H DI‐D in suppressing pain sensation, we tested the V_H_H DI‐D on a Na_v_1.7‐dependent pain model (OD1 pain model) in mice. Scorpion *α*‐toxin OD1, isolated from the venom of the Iranian yellow scorpion, is a 65‐residue C‐terminally amidated polypeptide. OD1 is a potent modulator of Na_v_1.7 (EC_50_ 4.5 nm), with little effect on Na_v_1.3 and Na_v_1.5 (EC_50_ > 1 µm) and no effect on Na_v_1.2 and Na_v_1.8.^[^ ^35^ ^]^ Intraplantar injection of OD1 in the hind paw of mice induces spontaneous pain behaviors as evidenced by licking, flinching, lifting and shaking of the injected hind paw. These behaviors are dose dependent and develop rapidly, occurring soon after injection, and persist for up to 40 min after injection.^[^ ^36^ ^]^ One major advantage of this model is that the peptide is delivered directly to the terminals of peripheral sensory neurons in the skin, allowing simple quantification of unilateral pain behaviors while avoiding systemic adverse effects. We tested the injection of different concentrations of OD1 in the dorsal side of the hind paw (Figure S7, Supporting Information). Twenty minutes after the injection, 22.5 pmol of OD1 evoked a cumulative response of 200 s (Figure S7, Supporting Information), in accordance to what has been previously reported in literature.^[^ ^21^ ^]^ Thus, we chose this concentration to test our compounds.

**Figure 4:**
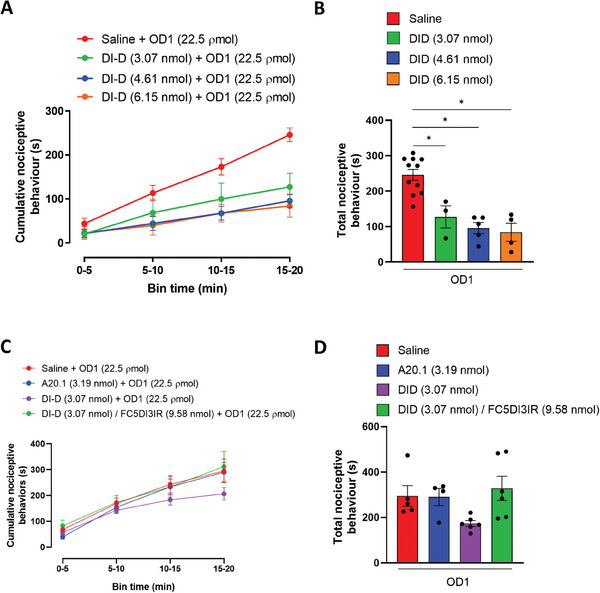
Effect of V_H_H DI‐D and DI‐D/FC5‐DIE3IR competition in the OD1‐induced mouse model of Na_v_1.7‐mediated pain. A) Intraplantar administration of V_H_H DI‐D (3.07, 4.61, and 6.15 nmol) reversed spontaneous pain behaviors in mice evoked by OD1 in a concentration‐dependent manner. OD1 was injected 60 min after V_H_H DI‐D administration and pain behaviors, as evidenced by licking, flinching, lifting and shaking of the injected hind paw (quantified in 5 min intervals). B) Total cumulative positive nociceptive behaviors during the 20 min study in the presence of saline or increasing concentrations of V_H_H DI‐D in the OD1 model. C) Competition study using FC5‐DIE3IR, the recombinant protein used as an immunogen for the generation of the Na_v_1.7 single domain antibodies. DI‐D / FC5‐DIE3IR were pre‐mixed 30 min prior to injection. OD1 was injected 60 min after saline, V_H_H DI‐D, DI‐D/FC5‐DIE3IR or A20.1 (negative control) administration and pain behaviors were quantified in 5 min intervals. D) Total cumulative positive nociceptive behaviors during the 20 min study in the presence of saline, V_H_H DI‐D, DI‐D/FC5‐DIE3IR or A20.1 in the OD1 model. Data are shown as mean ± SEM of 4 – 6 mice per group. *p < 0.05 versus saline + OD1.

We tested the effect of the pre‐injection of 50, 75, and 100 µg V_H_H DI‐D (3.07, 4.61, and 6.15 nmol, respectively) on the OD1‐induced behavior (Experimental Section). At all tested concentrations, V_H_H DI‐D reduced the pain behaviors induced by the injection of OD1 (**Figure** **4**A,B). Twenty minutes post‐injection, the total cumulative positive behavior was significantly (*p* < 0.05) reduced by DI‐D (50 µg, 127.27 ± 31.14 s; 75 µg, 95.62 ± 15.72 s; 100 µg, 83.91 ± 25.43 s) when compared to saline (245.97 ± 15.23 s; Figure 4B). These data suggest that, to reduce pain behavior in the OD1 mouse model, V_H_H DI‐D binds to the Na_v_1.7 channels (this is also confirmed by selectivity studies in V_H_H DI‐D selectivity section).

To further verify the binding of V_H_H DI‐D to Na_v_1.7, we performed a competitive study by injecting in the right hind paw the V_H_H DI‐D together with FC5‐DIE3IR, the recombinant protein used as immunogen for the generation of the Na_v_1.7 single domain antibodies. DI‐D (50 µg or 3.07 nmol) and FC5‐DIE3IR (200 µg or 9.58 nmol) were mixed at the ratio of 1:4 (DI‐D/FC5‐DIE3IR, gr gr^−1^) 30 min before injection. DI‐D/FC5‐DIE3IR had no effect in reducing the time mice spend in pain behaviors (311.68 ± 58.57 s, n = 6; Figure 4C,D), suggesting that the binding of DI‐D to the recombinant protein FC5‐DIE3IR prevents the binding of V_H_H DI‐D to the Na_v_1.7 channel.

**Figure 5:**
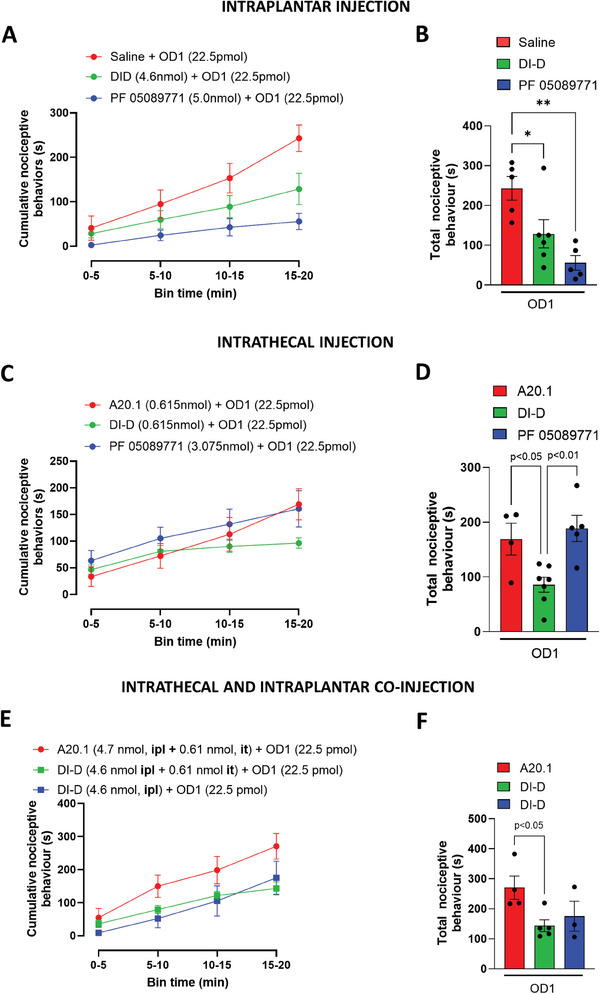
Effect of V_H_H DI‐D administered by intraplantar, intrathecal and, intraplantar and intrathecal routes in the OD1‐induced mouse model of Na_v_1.7‐mediated pain. A) Effect of intraplantar administration of saline, V_H_H DI‐D (4.61 nmol) or PF05089771 (5 nmol) on spontaneous pain behaviors in mice evoked by OD1. OD1 was injected 30 min after test compound administration and pain behaviors, as evidenced by licking, flinching, lifting, and shaking of the injected hind paw were quantified in 5 min intervals. B) Total cumulative positive nociceptive behaviors during the 20 min study in the presence of saline, V_H_H DI‐D (4.61 nmol) or PF05089771 (5 nmol) in the OD1 model. C) Effect of intrathecal administration of V_H_H A20.1, V_H_H DI‐D (0.615 nmol) or PF05089771 (3.075 nmol) on spontaneous pain behaviors in mice evoked by OD1. OD1 was injected 30 min after test compound administration and pain behaviors were quantified in 5 min intervals. D) Total cumulative positive nociceptive behaviors during the 20 min study in the presence of V_H_H A20.1, V_H_H DI‐D (4.61 nmol) or PF05089771 (5 nmol) in the OD1 model. E) Effect of intraplantar (ipl) and intrathecal (it) co‐administration of V_H_H A20.1 or V_H_H DI‐D (ipl, 4.6 nmol; it, 0.615 nmol,) and comparison with V_H_H DI‐D administered by intraplantar route only on spontaneous pain behaviors in mice evoked by OD1. OD1 was injected 30 min after test compound administration and pain behaviors were quantified in 5 min intervals. F) Total cumulative positive nociceptive behaviors during the 20 min study of V_H_H A20.1 and V_H_H DI‐D co‐injected by intraplantar and intrathecal route and comparison with V_H_H DI‐D injected by intraplantar route only in the OD1 model. Data are shown as mean ± SEM of 4–6 mice per group. *p < 0.05 versus saline + OD1 or saline + A20.1 + OD1.

The Na_v_1.7 channel is expressed in nociceptive neurons (DRG) of the PNS. Nociceptive stimuli (i.e., injury or inflammation) are initiated at peripheral receptors, and then transduced at peripheral termini by Na_v_ dependent action potentials. Na_v_1.7 channels are also present in the central axonal projections of DRG neurons and their presynaptic terminals within the dorsal horn of the spinal cord, facilitating impulse invasion or evoked release of neurotransmitters.^[^ ^9^ ^]^ We thus evaluated if the functional in vitro effect of V_H_H DI‐D on deactivation current decay translated into a functional in vivo effect in the OD1 pain model by injecting the V_H_H DI‐D peripherally (intraplantar) and within the dorsal horn of the spinal cord (intrathecal). We tested the possible synergistic effect of Na_v_1.7 channels inhibition at peripheral endings and DRG by performing a co‐administration of V_H_H DI‐D by intraplantar and intrathecal routes (**Figure** **5**A–F). In order to avoid the effect of excessive rounds of anaesthesia, in this set of experiments, test compound pre‐injections were performed 30 min prior to OD1 administration.

Figure 5A,B shows that 4.6 nmol of V_H_H DI‐D was effective (*p* < 0.05) in reducing OD1‐induced pain behaviors (128.65 ± 35.44 s, n = 6) when injected via intraplantar route 30 min before OD1, compared to saline (242.93 ± 29.90 s, n = 5). The Na_v_1.7 high‐affinity small molecule PF 05089771^[^ ^37^, ^38^ ^]^ was also effective in this model (55.81 ± 18.43 s, n = 5).

**Figure 6:**
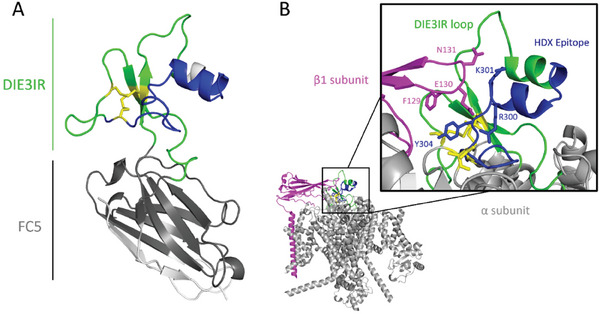
HDX‐MS protection highlights V_H_H DI‐D epitope within DIE3IR. A) Projection of HDX findings on a homology model of the FC5‐DIE3IR immunogen based a known DIE3 loop structure (PDB ID 6J8G). Regions where a significant change in HDX (> 2 SD cut‐off, *p*‐value 0.05) was measured upon binding of V_H_H DI‐D across at least two overlapping peptides are shown in blue, non‐significant changes are shown in green and grey for the DIE3IR loop and FC5 scaffold domains, respectively. Residues with missing sequence coverage are shown in white. Disulfide bonds are shown as yellow sticks. B) Overall view of HDX overlayed on channel architecture based on a recent cryo‐EM structure of the human hNa_v_1.7 channel (PDB 7W9K).^[^ ^28^ ^]^ Structural regions are highlighted, including the *α*‐subunit (grey), *β*1 subunit (magenta), and the DIE3IR loop (green) with its HDX‐mapped V_H_H DI‐D epitope (blue). Key interacting residues between *a* and *b* subunits of the hNa_v_1.7 channel are shown as sticks.

When administered by intrathecal route, V_H_H DI‐D (0.615 nmol) significantly reduced OD1‐induced nociceptor behaviour (96.45 ± 9.77 s, n = 6) compared to an equimolar concentration of A20.1 (169.09 ± 29.13 s, n = 4; Figure 5C). Surprisingly, when injected directly into the spinal cord, PF05089771 had no effect on OD1 response (160.71 ± 34.04 s, n = 6; **Figures** 5C and **6**D).

We then attempted to block Na_v_1.7 channels at the peripheral endings and in the spinal cord by injecting V_H_H DI‐D via intraplantar and intrathecal routes 30 min prior to OD1 administration. Figure 5E shows that V_H_H DI‐D (4.6 nmol intraplantar and 0.61 nmol intrathecal; 143.20 ± 19.80 s, n = 5) significantly (*p* < 0.05) reduces OD1‐mediated nociceptive behavior when compared to equimolar concentrations of A20.1 (270.49 ± 38.92 s, n = 4). Interestingly, no significant additional effect was observed when compared to V_H_H DI‐D injected by intraplantar route alone (Figure 5E,F).

Taken together, these data suggest that V_H_H DI‐D successfully blocks pain transmission in vivo via Na_v_1.7 channels, further supporting its development as a novel analgesic candidate for pain applications.

### 2.5 Identification of the hNa_v_1.7 Epitope for V_H_H DI‐D

To map the V_H_H DI‐D binding profile within DIE3IR we used differential Hydrogen Deuterium exchange Mass Spectrometry (**HDX‐MS**). This method provides a snapshot of the changes in conformational stability imparted by binding events by tracking the exchange of amide protons for deuterium, and is a powerful tool for mapping antibody based‐interactions.^[^ ^39^ ^]^ HDX‐MS (Experimental Section) was performed by comparing the extent of deuteration between V_H_H DI‐D bound and unbound forms of FC5‐DIE3IR. Overall, deuteration was measured for 41 peptides covering 81% of the sequence of the entire FC5‐DIE3IR, including 94% coverage of the grafted DIE3IR domain (Figure S8, Supporting Information). Full HDX‐MS kinetic measurements are shown in Table S5 (Supporting Information). First, no significant changes in deuteration were observed outside of the DIE3IR domain, reaffirming that V_H_H DI‐D binds the grafted DIE3IR domain. While it is challenging to measure changes in HDX for weak binders,^[^ ^40^ ^]^ we clearly observed perturbations in the underlying protein dynamics in response to V_H_H DI‐D. Decreases in HDX, which are the result of protection from exchange at an antibody‐antigen interface and/or conformational stabilization, in residues Asn291 – Phe317 were observed (Figure 6A). This binding profile is adjacent to the central pore and partially overlaps with the *β*1‐Na_v_1.7 interface (Figure 6B, magenta).^[^ ^28^ ^]^ The hNa_v_1.7 sequence 291‐NTLESEEDFRKYFY‐304, covering a majority of the V_H_H DI‐D HDX binding profile has no homology with any other member of the Na_v_ channels in human (taxid: 9606; Table S6, Supporting Information). Overall, this analysis suggests that the probability that V_H_H DI‐D acts on others subtypes of the Na_v_ family other than the Na_v_1.7 is very low.

### 2.6 DI‐D Selectivity

Compared with small molecules, mAbs and sdAbs are known to have an exquisite target selectivity and affinity toward their antigen by recognizing a specific aa sequence. To evaluate the selectivity of V_H_H DI‐D for hNa_v_1.7 we analyzed potential cross‐reactive sequences using the global sequence alignment method BLAST.^[^ ^41^ ^]^

First, we used as query sequence the 70 aa of DIE3IR used to generate the antigen (KHKCFRNSLENNETLESIMNTLESEEDFRKYFYYLEGSKDALLCGFSTDSGQCPEGYTCVKIGRNPDYGY; Table S6, Supporting Information). This analysis indicated that DIE3IR has a 60% identity (42 / 70 aa) with hNa_v_1.2. The homologies of the V_H_H DI‐D epitope to the other human Na_v_s are too low to be significant (Table S6, Supporting Information). Human Na_v_1.4 has a shorter extracellular loop (39 aa) than hNa_v_1.7. Even if they share 29 of the 39 aa, these aa are not part of the 14 aa epitope (Figure 6; Table S6, Supporting Information). Human Na_v_1.1 and hNa_V_1.3 share 51% and 46% of the 70 aa sequence, however there is no significant identity in the epitope region (Table S6, Supporting Information). Particular attention was put on the sequences of Na_v_1.8 and Na_v_1.9 (human, rat and mouse) and BLAST analysis revealed no homology.

Second, we used as query sequence the NTLESEEDFRKYFY identified by HDX‐MS as the epitope binding the V_H_H DI‐D (Figure 6B). This analysis indicated that this sequence has no identity whatsoever with any other Na_v_ isoform than hNa_v_1.7 (Table S6, Supporting Information).

To further evaluate V_H_H DI‐D selectivity, we tested it on the hNa_v_ subtypes with the highest aa identity within the DIE3IR using SyncroPatch 384PE. We used HEK293 cells overexpressing hNa_v_1.1 (51% identity) and CHO cells overexpressing hNa_v_1.2 (60% identity) (Experimental Section). We also tested V_H_H DI‐D on the cardiac channel isoform hNa_v_1.5. In in vitro patch‐clamp experiment using SyncroPatch 384 PE and HEK293‐hNa_v_1.1, CHO‐hNa_v_1.2 and HEK293‐hNa_v_1.5, V_H_H DI‐D had no effect on the amplitude and kinetics of the evoked currents (Figure S9, Supporting Information). Overall, these data showed that V_H_H DI‐D is selective only for the subtype 1.7 of the hNa_v_ family.

### 2.7 Biodistribution and Pharmacokinetic of V_H_H DI‐D

**Figure 7:**
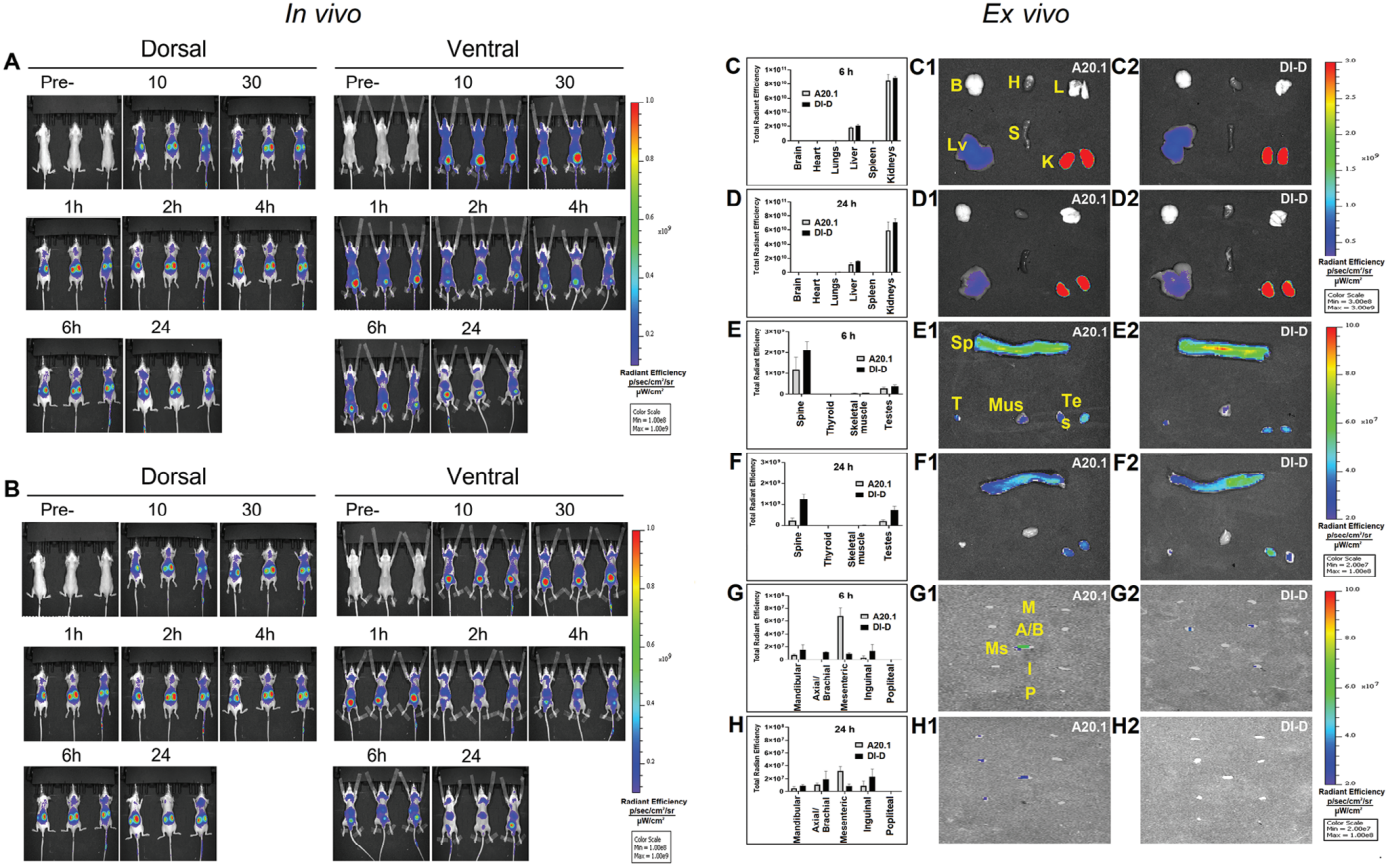
In vivo biodistribution of V_H_H A20‐CF770 or V_H_H DI‐D‐CF770 intravenously injected (0.4 mg kg^−1^) in naïve mice. A) In vivo dorsal (left) and ventral (right) images of the whole mouse body at various time points (10 min, 30 min 1 h, 2 h, 4 h, 6 h and 24 h) after intravenous injection of V_H_H A20.1‐CF770. B) In vivo dorsal (left) and ventral (right) images of the whole mouse body at various time points (10 min, 30 min 1 h, 2 h, 4 h, 6 h and 24 h) after intravenous injection of V_H_H DI‐D‐CF770. C–H) Ex vivo tissue biodistribution of V_H_H A20.1‐CF770 and V_H_H DI‐D‐CF770 intravenously injected (0.4 mg kg^−1^) in naïve mice. (C, D) Bar graph illustrating the total radiant efficiency in various organs (brain, heart, lung, liver, spleen kidney) ex vivo at 6 h (C) and 24 h (D) after intravenous injection of V_H_H A20.1‐CF770 or V_H_H DI‐D‐CF770, followed by saline perfusion and animal sacrifice. Representative ex vivo optical images of various organs (brain, heart, lung, liver, spleen, kidney) at 6 h (C1, C2) and 24 h (D1, D2) after intravenous injection of V_H_H A20.1‐CF770 or V_H_H DI‐D‐CF770, followed by saline perfusion and animal sacrifice. (E, F) Bar graph illustrating the total radiant efficiency in more difficult to access organs (spine, thyroid, skeletal muscle, testes) ex vivo at 6 h (E) and 24 h (F) after intravenous injection of V_H_H A20.1‐CF770 or V_H_H DI‐D‐CF770, followed by saline perfusion and animal sacrifice. Representative ex vivo optical images of various organs (spine, thyroid, skeletal muscle, testes) at 6 h (E1, E2) and 24 h (F1, F2) after intravenous injection of V_H_H A20.1‐CF770 or V_H_H DI‐D‐CF770, followed by saline perfusion and animal sacrifice. (G, H) Bar graph illustrating the total radiant efficiency in various lymph nodes ex vivo at 6 h (G) and 24 h (H) after intravenous injection of V_H_H A20.1‐CF770 or V_H_H DI‐D‐CF770, followed by saline perfusion and animal sacrifice. Representative ex vivo optical images of various lymph nodes at 6 h (G1, G2) or 24 h (H1, H2) after intravenous injection of V_H_H A20.1‐CF770 or V_H_H DI‐D‐CF770, followed by saline perfusion and animal sacrifice. *Abbreviations*: Brain (B), Heart (H), Lung (L), Liver (Lv), Spleen (S), Kidney (K), Mandibular (M), Axial/Brachial (A/B), Inguinal (I), Mesenteric (Ms), Popliteal (P), Spine (Sp), Thyroid (T), Skeletal Muscle (Mus), Testes (Tes). Data is presented as mean ± SEM, n = 3 mice per group and time point.

In order to evaluate if V_H_H DI‐D accumulates in organs that express Na_v_1.7, in vivo optical imaging was used to determine the tissue biodistribution of V_H_H DI‐D‐CF770 compared to a negative V_H_H control, A20.1‐CF770 in mice. Animals injected with either V_H_H DI‐D‐CF770 or V_H_H A20.1‐CF770 show a persistence of CF770 fluorescence in the body for over 24 h in both dorsal and ventral scans. Full body in vivo imaging indicates a similar pattern of biodistribution for both V_H_Hs with a strong accumulation in kidneys (**Figure** **7**A,B, dorsal) and bladder (Figure 7A,B, ventral), followed by lower signals in the neck, armpit and liver regions (Figure 7A,B, ventral). For a typical V_H_H, kidney clearance is predominant due to the small size of the V_H_H and lack of a Fc subunit.^[^ ^42^ ^]^

At the end of the imaging protocol, 6 and 24 h after injection of the V_H_H DI‐D‐CF770 or V_H_H A20.1‐CF770, ex vivo fluorescence was quantified in organs (Figure 7C‐F) and lymph nodes (Figure 7G,H). Both V_H_H DI‐D‐CF770 and V_H_H A20.1‐CF770 show strong and similar accumulation in kidney and liver at 6 and 24 h time points. At 24 h, fluorescent signals for V_H_H DI‐D‐CF770 were fivefold and 3.5‐fold higher in spinal cord and testes, respectively, compared to V_H_H A20.1‐CF770. For lymph nodes at 6 h, V_H_H DI‐D‐CF770 has a higher accumulation in axial/brachial lymph nodes compared to V_H_H A20.1‐CF770, while V_H_H A20.1‐CF770 accumulates more (7.4‐fold at 6 h and 3.8‐fold at 24 h) in the mesenteric lymph node compared to V_H_H DI‐D‐CF770.

To evaluate the pharmacokinetic of V_H_H DI‐D, Wistar rats were administered with a single intravenous bolus of V_H_H DI‐D at 4 mg kg^−1^ dose and the V_H_H concentration was quantified in serum over a 24 h collection period using mass spectrometry (**MS**) selected reaction monitoring (**SRM**) analysis (see Experimental Section). The V_H_H concentration at the 24 h time point was below the multiple reaction monitoring (**MRM**) lower limit of quantification (**LLOQ**) and therefore was not included in the pharmacokinetic (**PK**) analysis. V_H_H DI‐D displayed a biphasic disposition with a clear distribution and an elimination phase (Figure S10, Supporting Information). The half‐life (3.3 ± 0.37 h) of the V_H_H DI‐D was longer than the reported half‐life values (<2 h) for other V_H_H in rodents.^[^ ^43^, ^44^, ^45^ ^]^

Overall, these data show that V_H_H DI‐D accumulated in the spinal cord and testes, but did not significantly accumulate in brain and heart, minimizing the possibility of causing a negative effect on neural and cardiac electrical conduction.

### 2.8 Analgesic Effect of V_H_H DI‐D in In Vivo Pain Models

We next evaluated if the functional in vitro effect of V_H_H DI‐D on deactivation current decay translated into a functional in vivo effect in animal pain models.

**Figure 8:**
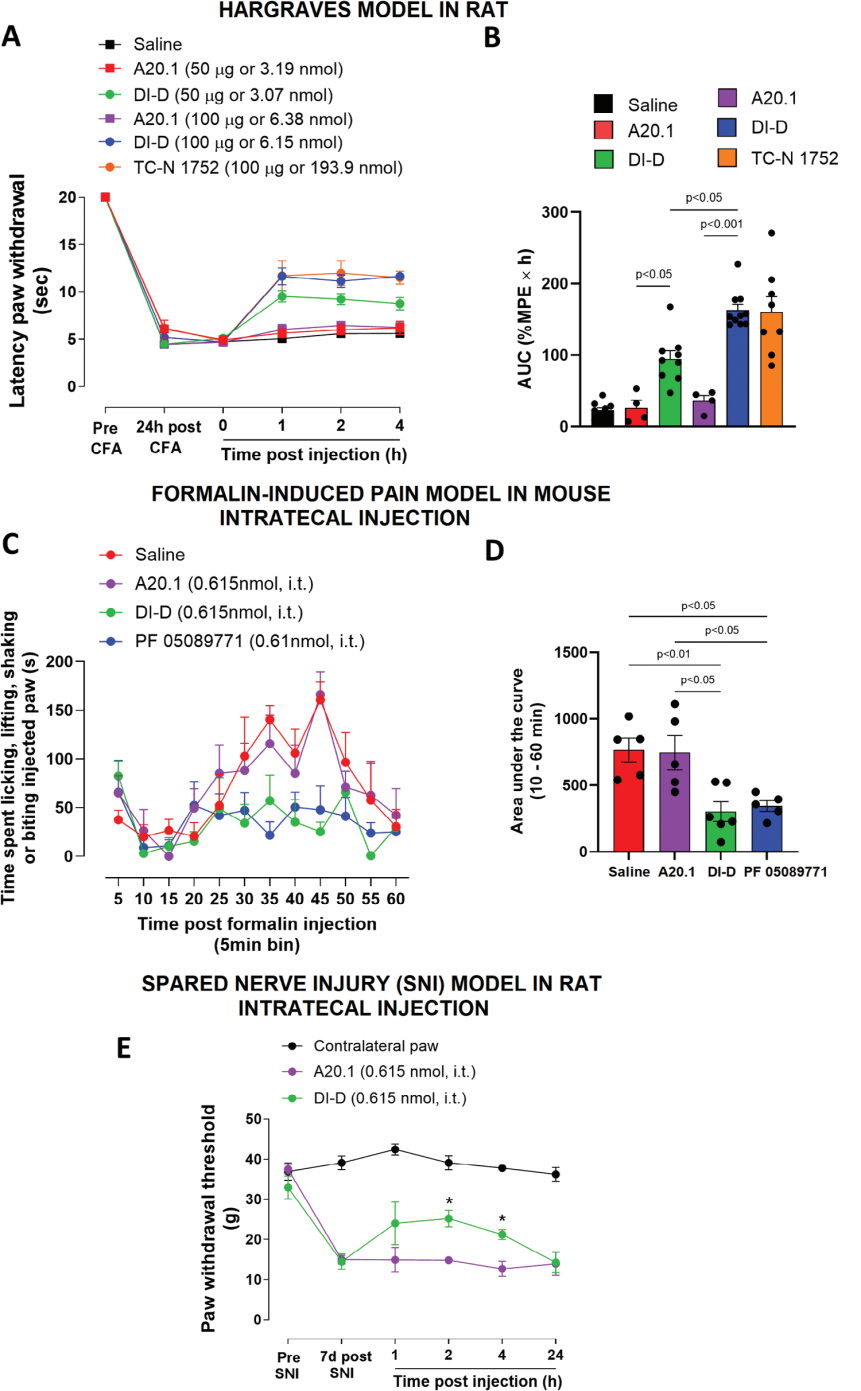
Efficacy of V_H_H DI‐D in pain models. A,B) V_H_H DI‐D‐induced reversal of thermal hyperalgesia in the Hargreaves model of inflammatory pain in rats. (A) Latency paw withdrawal of inflamed paw to a thermal stimulus was measured pre CFA injection, 24 h post CFA injection, and 0 (48 h post CFA injection), 1, 2, and 4 h after intraplantar injection of saline (50 µL), V_H_H DI‐D (50 and 100 µg, 3.07 nmol and 6.15 nmol, respectively in a volume of 50 µL), the negative control V_H_H A20.1 (50 and 100 µg, 3.19 nmol and 6.38 nmol, respectively in a volume of 50 µL) and the selective blocker for hNa_v_1.7 channels, TC‐N1752 (100 µg, 193.9 nmol in a volume of 50 µL). (B) Area under the curve (AUC) of the paw withdrawal latency from each group expressed as a percentage of maximal possible effect (%MPE) over time (h). Data are shown as mean ± SEM of 4–11 rats per group. C,D) Time course of the antinociceptive effect of V_H_H DI‐D in the formalin test in mice. (C) V_H_H DI‐D (0.615 nmol, in a volume of 5 µL), V_H_H A20.1 (0.615 nmol, in a volume of 5 µL), the positive control PF 05089771 (0.61 nmol, in a volume of 5 µL) or saline were injected by intrathecal route 30 min prior to formalin (2.5%) intraplantar injection. (D) Bar graph showing the area under the curve (AUC) of the positive behaviors observed from 10 to 60 min post formalin injection. Data are shown as mean ± SEM of 5–6 mice per group. E) Effect V_H_H DI‐D on mechanical allodynia induced by the spared nerve injury (SNI) model in rats. Time‐course of paw withdrawal threshold was measured by von Frey hair stimulation at 1, 2, 4, and 24 h after intrathecal injection of V_H_H DI‐D (0.615 nmol, in a volume of 5 µL) or the negative control V_H_H A20.1 (0.615 nmol, in a volume of 5 µL). Data are shown as mean ± SEM of 3–4 rats per group. *p < 0.05 versus V_H_H A20.1.

We tested V_H_H DI‐D in rats using the Hargreaves model of hyperalgesia (inflammatory pain model).^[^ ^46^ ^]^ V_H_H DI‐D was evaluated based on its ability to mediate reversal of hyperalgesia to thermal stimuli induced by complete Freund's adjuvant (**CFA**; see Experimental Section). The non‐noxious nature of the thermal stimulus applied to the hind paw was proved by the lack of response before CFA injection. In order to avoid tissue damage caused by the thermal stimulus, its duration was cut‐off at 20 s (**Figure** **8**A, pre CFA). Twenty‐four hours after the intraplanar injection of CFA, a significant decrease in latency of paw withdrawal was observed (saline 4.45 ± 0.30 s; n = 9; V_H_H DI‐D 50 µg 4.49 ± 0.22 s; n = 9; V_H_H DI‐D 100 µg 5.18 ± 0.49 s; n = 11; V_H_H A20.1 50 µg 6.67 ± 0.93 s; n = 4; V_H_H A20.1 100 µg 4.46 ± 0.25 s; n = 4; TC‐N 1752 100 µg 6.19 ± 0.39 s; n = 8, Figure 8A, 24 h post CFA). We evaluated the ability of V_H_H DI‐D to mediate reversal of hyperalgesia 48 h post CFA injection when the responses to thermal stimuli were consistently reduced to 5 s in all animals (Figure 8A; t = 0; saline 4.74 ± 0.21 s; n = 9; V_H_H DI‐D 50 µg 5.10 ± 0.19 s; n = 9; V_H_H DI‐D 100 µg 4.71 ± 0.23 s; n = 11; V_H_H A20.1 50 µg 4.93 ± 0.21 s; n = 4; V_H_H A20.1 100 µg 4.69 ± 0.23 s; n = 4; TC‐N 1752 100 µg 4.81 ± 0.17 s; n = 8). We compared the effect of intraplantar injection of V_H_H DI‐D (50 and 100 µg, 3.07 nmol and 6.15 nmol, respectively) with the negative controls saline and V_H_H A20.1 (50 and 100 µg, 3.19 nmol and 6.38 nmol, respectively), and with the selective hNa_v_1.7 blocker TC‐N1752 (100 µg, 193.9 nmol). TC‐N1752 is a small molecule which has been reported to be efficacious in pain animal models.^[^ ^47^ ^]^ In Figure 8A the time‐course and concentration‐response of the effects of saline, V_H_H DI‐D, A20.1 and TC‐N1752 are shown. V_H_H DI‐D significantly increased the latency paw withdrawal time at both 50 µg (1 h, 9.52 ± 0.60 s; 2 h, 9.24 ± 0.58 s; 4 h, 8.74 ± 0.65 s; n = 9) and 100 µg (1 h, 11.64 ± 0.88 s; 2 h, 11.13 ± 0.67 s, and 4 h, 11.65 ± 0.40 s; n = 11) when compared to the negative controls saline (1 h, 5.06 ± 0.30 s; 2 h, 5.58 ± 0.29 s; 4 h 5.60 ± 0.22 s; n = 9) and A20.1 (50 µg: 1 h, 5.63 ± 0.47 s; 2 h, 6.01 ± 0.52 s; 4 h 6.20 ± 0.71 s; n = 4; and 100 µg: 1 h, 6.01 ± 0.54 s; 2 h, 6.46 ± 0.42 s; 4 h 6.24 ± 0.51 s; n = 4). Notably, V_H_H DI‐D injected at 100 µg displayed a similar effect compared to TC‐N1752 (100 µg: 1 h, 11.72 ± 1.58 s; 2 h, 11.98 ± 1.30 s; 4 h, 11.51 ± 0.69 s; n = 8). Analysis of the Area Under the Curve (**AUC**) of the percentage of the Maximum Possible Effect over time (**%MPE** x h; Figure 8B; Table S4, Supporting Information) showed a concentration dependent analgesic effect of V_H_H DI‐D (94.85 ± 11.31 and 162.90 ± 8.22, at 50 and 100 µg, respectively). In addition, V_H_H DI‐D showed a similar effect compared to the Na_v_1.7 selective blocker TC‐N1752 (160.36 ± 21.46).

Next, we tested whether V_H_H DI‐D affects the behavioral responses to the chemical irritant formalin in mice. Formalin was injected into the dorsal surface of the hind paw of mice under light isoflurane anaesthesia (see Experimental Section).^[^ ^48^ ^]^ V_H_H DI‐D (0.615 nmol, in a volume of 5 µL), V_H_H A20.1 (0.615 nmol, in a volume of 5 µL), PF 05089771 (0.61 nmol, in a volume of 5 µL) or saline were administered by intrathecal route and the time spent licking, lifting, shacking or biting the injected paw over the next 60 min was counted (AUC: V_H_H DI‐D, 302.9 ± 74.4 s, n = 6; V_H_H A20.1, 745.1 ± 129.0 s, n = 5; PF 05089771, 344.7 ± 39.7 s, n = 5; saline, 765.5 ± 90.2 s, n = 5;). In Figure 8C the time‐course of the effects of saline, V_H_H DI‐D, V_H_H A20.1 and PF 05089771 on formalin‐induced behaviors are shown. Formalin is known to cause a biphasic response with an early, acute phase and a late inflammatory phase.^[^ ^49^ ^]^ Since we performed formalin injection under anaesthesia due to animal welfare concerns, in Figure 8D we included only the quantification of pain responses ranging from 10 to 60 min post formalin injection (Figure 8D, AUC 10–60 min) to avoid the interference of the anesthesia on our measures. A significant reduction in formalin‐induced behaviors is observed following administration of V_H_H DI‐D, further supporting its analgesic properties.

Finally, we tested whether V_H_H DI‐D had an effect in the spared nerve injury (**SNI**) model in rat (see Experimental Section), a neuropathic pain model that causes robust and long‐lasting mechanical allodynia in the lateral portion of the affected limb.^[^ ^49^ ^]^ In this model, a significant change is observed in the withdrawal threshold of the ipsilateral paw following stimuli delivered using Von Frey monofilaments (see Experimental Section; Figure 8E), whereas no changes were observed in the contralateral hind limb.^[^ ^49^ ^]^ Figure 8E shows that the intrathecal injection of V_H_H DI‐D (0.615 nmol, in a volume of 5 µL), but not the negative control A20.1 (0.615 nmol, in a volume of 5 µL), induced a significant change in mechanical allodynia up to 4 h post injection (1 h, contralateral paw 42.4 ± 1.3 g, n = 4; V_H_H DI‐D 24.0 ± 4.6 g, n = 4; V_H_H A20.1 14.9 ± 2.7 g, n = 3; 2 h, contralateral paw 39.1 ± 1.7 g, n = 4; V_H_H DI‐D 25.2 ± 1.04 g, n = 4; V_H_H A20.1 14.8 ± 0.2 g, n = 3; 4 h contralateral paw 37.8 ± 0.8 g, n = 4; V_H_H DI‐D 21.2 ± 0.6 g, n = 4; V_H_H A20.1 12.7 ± 1.1 g, n = 3). The paw withdrawal threshold returned to baseline after 24 h (contralateral paw 36.2 ± 1.6 g, n = 4; V_H_H DI‐D 14.3 ± 1.3 g, n = 4; V_H_H A20.1 13.9 ± 1.6 g, n = 3) which is not surprising given the short half‐life of V_H_Hs.

Overall, these results suggest that targeting Na_v_1.7 with V_H_H DI‐D successfully reduces the perception of pain sensation caused by different pain stimuli (thermal, mechanical and chemical), further supporting the development of V_H_H DI‐D as a novel therapeutic compound for pain applications.

### 2.9 V_H_H DI‐D Mechanism of Action

**Figure 9:**
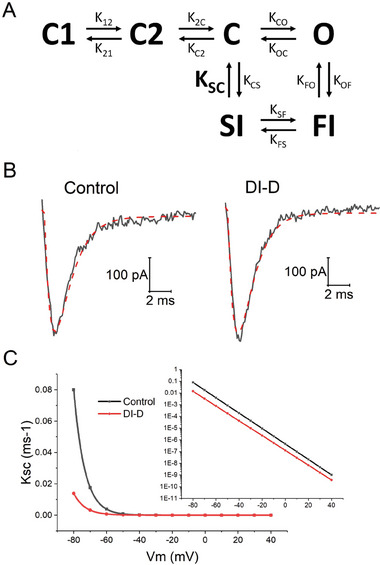
Kinetic model and simulation of Na_v_1.7 currents. A) Kinetic model of Na_v_1.7 channel. The kinetic scheme consists of three closed states (C1, C2, and C), one open state (O), one fast inactivated state (FI) and one slow inactivated state (SI). B) Na_v_1.7 current curves. The solid lines represent the Na_v_1.7 currents recorded in patch clamp in control and in presence of DI‐D following a voltage step at −30 mV from a Vm of −120 mV. The red dashed lines represent the currents simulated according to the kinetic scheme in A. C) Voltage dependence of the rate constant K_SC_ of the transition from the SI to the C. K_SC_ values were calculated after the micro‐reversibility relationship within the C – O – FI – SI loop. The semi‐log plot in the inset shows that in the range of voltages between −80 and +40 mV, the transition K_SC_ is slower in presence of V_H_H DI‐D.

To explain how the effect of V_H_H DI‐D on the deactivation kinetics of Na_v_1.7 could translate into a reduction of pain perception observed during in vivo experiments, we utilized the kinetic model showed in **Figure** **9**A, a 6‐state kinetic scheme simplified from Gurkiewicz and collaborators.^[^ ^50^ ^]^ We focused on the observation obtained during in vitro experiments that the deactivation time constant of tail current decay increases in the presence of V_H_H DI‐D. Our hypothesis is that this also happens for the rate constant from slow inactivated (**SI**) to close (**C**, deactivated) states. This kinetic model was used to simulate the Na_v_1.7 currents elicited by different voltage pulses (−30, −10, 0, and +20 mV) before (control) and 5 min after application of 2 µm V_H_H DI‐D. The rate constants of transitions between different kinetic states were adjusted to best fit the currents recorded in both experimental conditions. The only exceptions were: i) the rate constant of the transition from SI to C (**K_SC_**), which was calculated using the micro‐reversibility relationship within the C – **O** (open) – **FI** (fast inactivation) – SI loop, and ii) the rate constant from O to C (**K_OC_**) that was obtained from patch‐clamp measurements (Figure 9A). Figure 9B shows the recorded currents (solid lines) and the simulated currents (dashed lines) at −30 mV in control and in presence of V_H_H DI‐D. Based on simulations (repeated on six different cells from four different experiments), the K_SC_ rate constant was calculated for the 4 voltage points studied (−30, −10, 0, and +20 mV). To determine the voltage dependence of K_SC_, data were then fitted using a single exponential function. The values for the voltages between −80 and +40 mV were then extrapolated (Figure 9C). As shown in the inset semi‐log plot of Figure 9C, in presence of V_H_H DI‐D the transition from SI to C is slower at all voltages. At −80 mV, the K_SC_ was 0.80 ± 0.78 ms^−1^ in control and 0.13 ± 0.25 ms^−1^ after application of V_H_H DI‐D (n = 6, *p* = 0.037, paired t‐test).

A slower deactivation kinetic is often reported in Na_v_1.7 channels of erythromelalgia patients^[^ ^51^, ^52^, ^53^, ^54^ ^]^ and is also often associated with Na_v_1.7 gain‐of‐function. In in vivo experiments, V_H_H DI‐D exhibited an analgesic effect which should correlate with a loss‐of‐function rather than with a gain‐of‐function, as seen in erythromelalgia. It is important to note that the hyperalgesia in erythromelalgia patients results from a complex ensemble of multiple modifications in the kinetic parameters of the mutated channel.^[^ ^51^, ^52^, ^53^, ^54^ ^]^ We hypothesize that the loss of function results from the fact that V_H_H DI‐D exerts an effect not only on the deactivation kinetic from O to C (measures obtained in patch‐clamp experiments), but also from SI to C. We suggest that the overall deactivation process, whether occurring from O or from SI, is slowed by the binding of V_H_H DI‐D to the channel. The deactivation process occurs when the membrane potential repolarizes to strongly negative voltages after the falling phase of the action potential and involves the return of the voltage sensor from the active (outwardly oriented) position to the resting (across the membrane) position. This conformational movement of the voltage sensor has been shown to entail a rearrangement of all the transmembrane domains of the channel.^[^ ^55^ ^]^ Hence, the binding of the V_H_H DI‐D to the DIE3IR loop, closely connected to the pore lining domains of the channel, may slow this movement, thus decreasing the number of channels ready to open for the next action potential. Finally, the role played by the *β*1 subunit into hNa_v_1.7 channel regulation^[^ ^56^ ^]^ and the apparent juxtaposition of the putative V_H_H DI‐D and *β*1 binding site on the DIE3IR loop calls for further studies to uncover a plausible interplay between the V_H_H DI‐D and *β*1 subunit to channel deactivation.

### 2.10 Effect of V_H_H DI‐D on DRG Neurons

Na_v_1.7 is an integrator/sensor of small depolarizations initiating the nociceptive signaling in DRG neurons.^[^ ^57^ ^]^ Small‐diameter (<30 µm) DRG neurons correspond to nociceptors. To verify weather the mechanism of action we described using our kinetic model can cause an actual reduction in the firing of DRG neurons, we recorded cryo‐preserved rat DGR neurons (≈20 µm of diameter) (see Experimental Section) using manual whole‐cell patch‐clamp in current clamp mode and measured the effect of V_H_H DI‐D (2 µm) on their ability to elicit action potentials.

**Figure 10:**
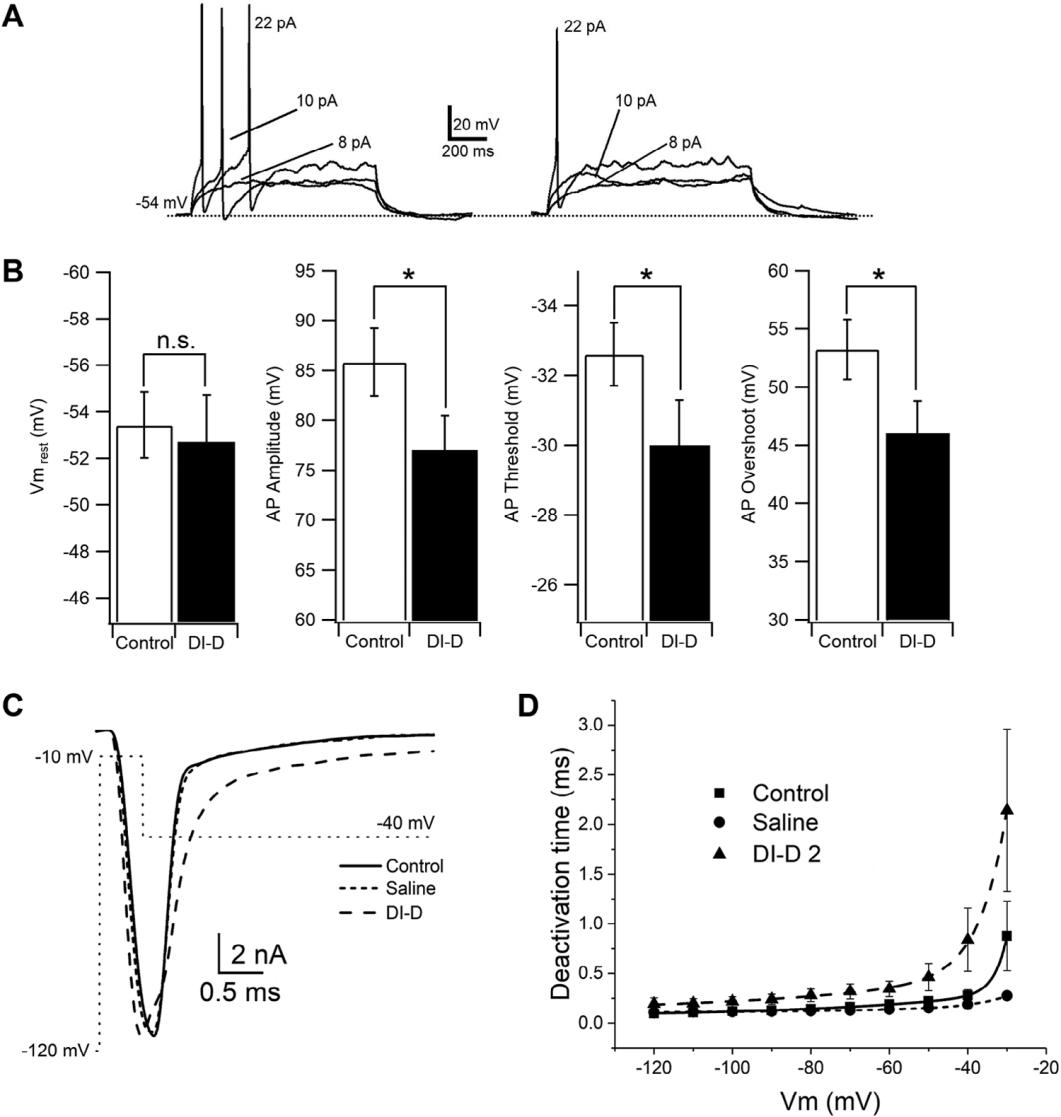
Effect of V_H_H DI‐D on the ability of DRG neurons to elicit action potentials A,B) and on the deactivation kinetics of DRG Na_v_ current C,D). (A) Action potentials (APs) were evoked from small (<20 µm in diameter) DRG neurons by injecting currents from Vm_rest_ in control (left) and in presence of V_H_H DI‐D 2 µm (right). (B) Histograms of values for Vm_rest_ (Control, −53.4 ± 1.41 mV; V_H_H DI‐D −52.7 ± 2.04 mV; n = 8, *p* = 0.3), AP amplitude (Control 85.82 ± 3.38 mV; V_H_H DI‐D 77.14 ± 3.47 mV, n = 8; *p* = 0.0018), AP threshold (Control, −32.56 ± 0.93 mV; V_H_H DI‐D, −30.2 ± 1.34 mV, n = 8; *p* = 0.003) and AP overshoot (Control, 53.35 ± 2.56 mV; V_H_H DI‐D 46.77 ± 3.47 mV; n = 8; *p* = 0.004) in control (white) and in presence of V_H_H DI‐D. n.s.: not significant. *Significant. (C) Example of deactivation tail currents with superimposed voltage‐clamp protocol. (D) Voltage dependence of the deactivation currents decay.

In presence of V_H_H DI‐D, DRG neurons needed a larger injection of current (rheobase: control, 22 ± 7.2 pA; DI‐D, 28 ± 7.5 pA, n = 8, *p* = 0.01) to be able to evoke their first action potential (**AP**; **Figure** **10**A). The ability of the neurons to elicit multiple APs was also reduced in presence of V_H_H DI‐D (Figure 10A). Figure 10B shows the effect of V_H_H DI‐D on the voltage membrane potential at rest (Vm_rest_), amplitude of AP, threshold of AP and overshoot of AP, respectively. V_H_H DI‐D significantly reduced the amplitude of AP, AP threshold and AP overshoot. Overall, these data support a reduced excitability of DRG neurons in presence of V_H_H DI‐D.

To verify that the effect of V_H_H DI‐D (2 µm) on the deactivation kinetic of the native rat Na_v_1.7 current in small‐diameter (<30 µm) DRGs, was similar to that of hNa_v_1.7 recorded in hNav1.7‐HEK 293 cells, we recorded Na_v_ currents from cryo‐preserved rat DGR neurons using manual whole‐cell patch‐clamp in voltage clamp mode (see Experimental Section). Small diameter rat DRG neurons express different types Na_v_ channels (mainly 1.7, 1.8, and 1.9).^[^ ^57^ ^]^ We did not use any specific Na_v_ blockers, consequently, the effect of V_H_H DI‐D could be considered underestimated. V_H_H DI‐D caused a significant increase in the time constant of deactivation current decay between −90 and −30 mV (Figure 10C). The time constants were 0.28 ± 0.06 ms (n = 4) in control, saline 0.19 ± 0.01 ms (n = 2) and 0.84 ± 0.31 ms (n = 3) in the presence of V_H_H DI‐D (*p* = 0.04) at ‐40 mV and 0.22 ± 0.04 ms (n = 4) in control, saline 0.15 ± 0.01 ms (n = 2) and 0.46 ± 0.13 ms (n = 3) in the presence of V_H_H DI‐D (*p* = 0.05) at −50 mV.

These data are in accordance with the ability of V_H_H DI‐D to reduce pain sensation.

## 3 Conclusion

Using a novel antigen presentation strategy aimed at maintaining the native 3D structure of an extracellular loop excised out of the full‐length hNa_v_1.7, we were able to isolate six V_H_H binders. One V_H_H showed functional activity, slowing the deactivation kinetics of the channels, reducing the ability for eliciting action potentials in nociceptors and reversing hyperalgesia in in vivo animal models. This V_H_H has the potential to be used for the development of a new therapeutic for pain indications. In addition, this new antigen presentation strategy could have a wider application in generating mAbs and sdAbs against other so‐called “difficult targets” such as ion channels, transporters and GPCRs.^[^ ^58^ ^]^

Doubts about Na_v_1.7 as a suitable target for pain arose when small molecules such as PF 05089771 failed in clinical trials. In 2016, Pfizer discontinued the development of PF 05089771 following insufficient efficacy in a Phase 2 trial against diabetic neuropathy.^[^ ^37^ ^]^ This therapeutic formed part of a series of molecules^[^ ^59^ ^]^ developed to target the VSD4 and provided the first series of Na_v_1.7 inhibitors with high selectivity over Na_v_1.5. However, no preclinical efficacy or pharmacodynamic data have been disclosed, and it is unclear what level of Na_v_1.7 inhibition was achieved in patients.^[^ ^38^ ^]^ Some controversy also arose from the suggestion that increased endogenous opioids in Na_v_1.7 knockout (**KO**) mouse were producing the insensitivity to pain.^[^ ^60^ ^]^ A very recent paper, using behavioral pharmacology and single‐cell RNA‐seq analysis in Na_v_1.7 KO mice showed that enkephalin overexpression, previously thought to drive analgesia on Na_v_1.7 KO mice, does not play any role in analgesia.^[^ ^61^ ^]^ In addition, using a tamoxifen‐inducible Na_v_1.7 KO mouse, which allows adult deletion of SCN9A, behavioral responses to most modalities of noxious stimuli were abolished with no need to recruit other systems, indicating that Na_v_1.7 blockade should be efficacious.^[^ ^62^ ^]^ Also, ablating SCN9A expression in all sensory neurons abolished only mechanical pain, inflammatory pain and reflex withdrawal responses to heat.^[^ ^63^ ^]^ Ablating SCN9A in both sensory and sympathetic neurons abolished pain sensations and recapitulated the pain‐free phenotype observed in humans with SCN9A loss‐of‐function mutations.^[^ ^63^ ^]^

Compared to small molecules, biologics (mAbs and sdAbs) have the potential for much greater selectivity. However, the majority of biologics developed against ion channels so far have been binders lacking functional efficacy. Ion channels possess multiple transmembrane regions with small extracellular loops, are difficult to purify (unstable antigens) and their functional epitopes are generally located inside the transmembrane pore. For these reasons, such targets are defined as “difficult”.^[^ ^58^ ^]^ It is also believed that targeting extracellular loops is not efficacious in producing blockers/inhibitors. However, the tarantula toxin ProTx‐II, a highly selective Na_v_1.7 inhibitor,^[^ ^64^ ^]^ does not bind the Na_v_1.7 pore region,^[^ ^65^ ^]^ demonstrating that targeting molecular regions outside the pore could be a successful strategy in identifying subtype‐selective compounds.

In order to develop sdAbs against hNa_v_1.7, we used a novel antigen presentation strategy that could be applicable to other transmembrane targets possessing extracellular loops of at least 40 amino‐acids – the size of some of the smallest folded protein domains found in nature.

The auxiliary *β* subunits of Na_v_s have multiple functions including the trafficking of the protein complex to the plasma membrane. All *β* subunits affect the channel properties of Na_v_1.7, although *β*1 and *β*2 are more commonly co‐expressed with the Na_v_1.7 *α* subunit for biophysical characterization.^[^ ^18^, ^19^ ^]^ The 3D structures of eukaryotic Na_v_ channels, including electric eel Na_v_1.4^[^ ^26^ ^]^ and the human Na_v_1.7^[^ ^19^, ^28^ ^]^ revealed that the *a*‐*b*1 interaction involves the immunoglobulin (Ig)‐like folded domain of *b*1 and part of the DIE3 extracellular loop of the *a* subunit (Figure 1D). Moreover, the region of the Ig‐like *b*1 domain equivalent to the specificity‐determinant CDR3 loop of antibody variable Ig domain makes direct contact with a portion of the epitope identified by HDX‐MS for the V_H_H DI‐D. We suggest that: i) the DIE3 is prone to interact with Ig‐like folded protein domains, thus justifying our selection of this loop for immunogen design; ii) regulation of channel activity by the *b* subunit^[^ ^18^ ^]^ via interaction with DIE3 could explain the functional activity of V_H_H DI‐D; iii) the correlation between binding affinity and functional activity of antibodies targeting the DIE3 loop is not straightforward due to the apparent partial overlap between the binding sites for *b* subunits and the epitope for V_H_H DI‐D. Indeed, V_H_Hs tested for function on hNa_v_1.7‐HEK293 cells using SyncroPatch 384 PE showed that their effect on Na_v_1.7 current did not necessarily correlate with the ELISA‐based binding profiles performed on cells expressing hNa_v_1.7.

HDX‐MS binding profiles showed structural perturbations upon binding of V_H_H DI‐D in a linear stretch of residues that span the region containing two short *α*‐helices of the DIE3IR domain, as well as the *β*1 binding site (PDB: 7W9K). The entire DIE3IR loop appears to be maintained by other amino‐acids which are distant in sequence but close by in 3D structure, by the proximal disulfide bonds (C275‐C322 and C315‐C330), and also the contact with several *β*1 subunit residues (inset, Figure 6B). While we cannot deduce the precise location of the epitope given the low‐resolution of the HDX data, we hypothesize that the epitope is partially conformational in nature. The high‐density of positively‐charged residues in CDR3 (QKRGEKKT) of V_H_H DI‐D suggests an interaction with a pore‐facing patch of negative charges (293‐ESEED‐298) that remain accessible in the *β*1‐Na_v_1.7 complex, favoring a pore‐facing epitope. Interestingly, this negatively‐charged surface patch is adjacent to residues R300, K301, and Y304 from the same HDX‐MS‐mapped epitope 291‐NTLESEEDFRKYFY‐304 which interact with residues E130, N131 and F129, respectively, from the CDR3‐like loop of *β*1 subunit (Figure 6B). While it is possible that *β*1 and V_H_H DI‐D are cooperative for binding to Na_v_1.7, we cannot rule out competition between V_H_H DI‐D and *β*1. Given the proximity of both binding sites and the role of *β*1 in inactivation of the channel,^[^ ^66^ ^]^ the interplay between V_H_H DI‐D and *β*1 warrants further investigation.

Selectivity is particularly relevant with regard to safety. Potential cross‐reactive sequences can be analyzed using global sequence alignment methods such as BLAST.^[^ ^41^ ^]^ A bioinformatics analysis indicated low homologies of the V_H_H DI‐D epitope to other human Na_v_s, which can be taken as a preliminary indication of antibody selectivity toward hNa_v_1.7. Pre‐incubation of V_H_H DI‐D together with FC5‐DIE3IR in OD1 pain model suggests that V_H_H DI‐D binds specifically to the Na_v_1.7 to exert its function. Importantly, V_H_H DI‐D did not affect the function of the hNa_v_1.1, hNa_v_1.2 and hNa_v_1.5 channels in vitro.

It could be speculated that in the OD1 pain model, pre‐treatment with the V_H_H DI‐D could potentially prevent OD1 from binding to Na_v_1.7. We exclude the possibility of competition for the same binding site between OD1 and V_H_H DI‐D. In fact, OD1 would bind to the S4 helix within the DIV VSD,^[^ ^67^ ^]^ whereas, as shown in HDX‐MS experiments, V_H_H DI‐D binds to a pore‐facing patch of negative charges between S5 and S6 on DI (Figure 6). However, we cannot rule out an eventual level of steric hinderance between the two molecules.

Na_v_1.7 is an integrator/sensor of small depolarizations initiating the nociceptive signaling in DRG^[^ ^57^ ^]^ and contributes to neuron excitability in the dorsal horn of the spinal cord.^[^ ^9^ ^]^ Targeting both DRG and dorsal horn neurons could be essential to reach a good level of analgesia. We have shown that intrathecal injection of V_H_H DI‐D significantly reduces pain responses in formalin and neuropathic pain models (Figure 8C–E). However, supplementing intraplantar with intrathecal injections of V_H_H DI‐D in OD1 model did not further reduce the nociceptive behavior. Interestingly, whereas the Na_v_1.7 small‐molecule inhibitor PF 05089771 failed to reduce pain behaviors in the OD1 model when injected intrathecally, it did have an effect in the formalin model. This is in agreement with a previous report showing ex vivo effects of PF 05089771 on the excitability of rat dorsal horn neurons.^[^ ^68^ ^]^

The low affinity of V_H_H DI‐D may contribute to the incomplete reversal of hyperalgesia, raising the possibility that a higher affinity variant of V_H_H DI‐D could completely reverse hyperalgesia. Efforts are underway to generate affinity‐matured version of V_H_H DI‐D followed by in vitro and in vivo experiments.

In our biodistribution studies, V_H_H DI‐D significantly accumulated in the spine, testes, and axial/brachial compared to V_H_H A20.1. A high presence of V_H_H DI‐D in kidney and liver is expected since these are the predominant clearance ways.^[^ ^42^ ^]^ The presence of V_H_H DI‐D in lung, axial/brachial and mandibular lymph nodes is explained by the presence of Na_V_1.7 in lung‐specific vagal sensory neurons and in jugular and nodose ganglia.^[^ ^69^ ^]^ Indeed, Na_v_1.7 blockers are used to inhibit persistent coughing, without having an effect on breathing. The presence of Na_v_1.7 in reproductive organs and lymph nodes is in accordance with a report showing that lymph nodes are innervated by nociceptors.^[^ ^70^ ^]^ In addition, the presence of V_H_H DI‐D in testes is probably due to the homology of the DIE3IR with the testis‐expressed protein 2 isoform X5 (Table S6, Supporting Information). Most importantly, the levels of V_H_H DI‐D are not significant in brain and heart, consequently V_H_H DI‐D should not exert any negative effect on neural and cardiac electrical conduction, supporting the high specificity for Na_v_1.7 and a safe organ distribution.

High‐affinity compounds binding to the resting state of VSDs have been developed as pain therapeutics.^[^ ^20^ ^]^ They work by preventing the voltage sensor from activating upon depolarization and keeping the channel in a closed state. However, these compounds have very limited analgesic effect.^[^ ^21^ ^]^ The V_H_H DI‐D showed a completely different mechanism of action that could overcome the analgesic ineffectiveness that compounds binding to VSDs had. We suggest that slower deactivation decreases the number of channels available to be open by depolarization during pain stimuli, which is consistent with a reduction of pain perception. This is further confirmed by the ability of V_H_H DI‐D to reduce the initiation of action potentials in nociceptors.

## 4 Experimental Section

### 3D‐Structure Modeling and Design of the FC5‐DIE3IR Immunogen

Molecular 3D structures for the V_H_H FC5 and the DIE3 loop of human Na_v_1.7 (hNa_v_1.7) channel *α* subunit were constructed by homology modeling using the MODELLER 9 program.^[^ ^71^ ^]^ The V_H_H FC5 model was based on the 1.34‐Å crystal structure of a homologous V_H_H with PDB ID 2 × 10.^[^ ^72^ ^]^ The CDR3 loop of the V_H_H FC5 model was further refined by Monte‐Carlo‐minimization conformational sampling,^[^ ^73^ ^]^ in which dihedral angles in the loop Gly99‐Tyr111 were sampled followed by energy minimization with the AMBER force field.^[^ ^74^, ^75^ ^]^ The Na_v_1.7 DIE3 loop model (amino‐acid residues Met268‐Thr346) was based on the 4‐Å cryo‐EM structure of the homologous electric eel Na_v_1.4 channel with PDB ID 5XSY.^[^ ^26^ ^]^ Sequence alignments to the respective templates used for homology modeling are given in Figure S1B (Supporting Information). The generated nanobody and DIE3IR models were then joined into a single polypeptide chain by structural manipulation in the Sybyl 8.1.1 program (Tripos, Inc. St. Louis, MO). The DIE3 loop terminal segments Met268‐Leu271 and Thr341‐Thr346, as well as the nanobody CDR3 inner region Ser102‐Leu107 were deleted, and the resulting fragments were manually positioned in order to allow polypeptide chain fusion with minimal distortion (below 1 Å from ideal amide‐bond geometry). The molecular structure of the resulting protein graft (the immunogen) was refined by energy minimization with the AMBER force‐field with harmonic constraints on side‐chain and main‐chain heavy atoms of 5 and 20 kcal mol^−1^ Å^−1^, respectively, and by allowing unconstrained minimization of two amino‐acid residues on either side of the two fusion sites. A structural model of the FC5‐DIE3IR immunogen based on the conformation of the DIE3 loop from the recently determined cryo‐EM structure of hNa_v_1.7 (PDB ID 6J8G)^[^ ^19^ ^]^ was constructed in a similar way and used retrospectively for analysis of epitope mapping data.

### FC5‐DIE3IR Protein Production

The codon‐optimized sequence encoding the human DIE3IR domain engrafted within the V_H_H FC5 in place of CDR3 region was synthesized (GenScript, Piscataway, NJ, USA) and cloned into the pTT5 vector. The equivalent sequences encoding for rat and mouse DIE3IR domain were also engrafted within the V_H_H FC5, sequenced and cloned as the human one.

The Chinese hamster ovary cell line expressing a truncated EBNA1 protein (CHO‐3E7; Figure S2A, Supporting Information)^[^ ^31^ ^]^ were grown in suspension in serum‐free FreeStyleTM F17 medium (Invitrogen, Carlsbad, CA, USA) supplemented with 0.1% Kolliphor P188 (Sigma‐Aldrich, St. Louis, Missouri, USA) and 4 mm Glutamine (Sigma–Aldrich, St. Louis, Missouri, USA). Cultures were maintained in 125 mL Erlenmeyer ventilated flasks shaken at 120 rpm in a humidified incubator at 37 °C with 5% CO_2_. Cell density and viability were determined using the Cedex Innovatis automated cell counter Cedex Analyzer (Roche, Basel, Switzerland) based on the trypan blue exclusion method.

Linear deacylated polyethylenimine Max (PEImax) was obtained from Polysciences (Warrington, PA, USA). A stock solution (3 mg mL^−1^) was prepared in ultrapure water, sterilized by filtration (0.2 µm), aliquoted and stored at 4 °C. Cells were diluted one day before transfection in fresh medium to a final concentration of 0.7 × 10^6^ cells mL^−1^. Cells were then transfected the following day, with a viability observed of greater than 99% at densities between 2.0 and 2.5 × 10^6^ cells mL^−1^ with 1 µg of plasmid DNA per mL of CHO culture. Plasmid DNA and PEImax were separately prepared in complete F17 medium. Plasmid DNA (pTT5‐ FC5‐DIE3IR) was diluted at 20 µg mL^−1^ in F17 medium, and an equivalent volume of F17 medium containing 100 µg mL^−1^ of 25 kDa PEImax was added. The polyplexes mixture was immediately vortexed and incubated for 5 min at room temperature prior to addition to the cells. Twenty‐four hours post‐transfection, cells were fed with peptone TN1 (0.5% w/v final), and the culture temperature was shifted to 32 °C.

Cell culture was centrifuged 20 min at 3000 g 10–12 days post‐transfection, with a viability observed > 60%. The supernatant was collected and loaded on a 5 mL MabSelect SuRe column (GE Healthcare) equilibrated in PBS. The column was washed with PBS and FC5‐DIE3IR was eluted with 100 mm citrate buffer pH 3.6. The fractions containing FC5‐DIE3IR were pooled and the citrate buffer was exchanged against PBS on Econo‐Pac 10DG columns (Bio‐Rad, cat# 732‐2010). Purified FC5‐DIE3IR was concentrated on Amicon Ultra 3 kDa and sterilized by passing through 0.2 µm filters, aliquoted, and stored at −80 °C.

### Intact Mass Spectrometry Analysis

FC5‐DIE3IR (10 µg at 0.5 mg mL^−1^ in 50 mm Tris‐HCl pH 7) was deglycosylated with 2 U PNGaseF (Sigma) overnight at 37 °C. Both deglycosylated and untreated FC5‐DIE3IR were analyzed by intact **LC‐MS** (liquid chromatography mass spectrometry) using an Agilent 1000 HPLC system coupled via an Ion Max electrospray source to an SYNAPT G2 Q‐TOF mass spectrometer (Waters). The samples (5 µg) were injected onto a 2.1 × 30 mm Poros R2 reverse phase column (Applied Biosystems) and desalted using a fast (3 min, 3 mL min^−1^) 0.1% formic acid aq/acetonitrile linear gradient (20–90% acetonitrile). The column and solvents were heated to 80 °C. The **HPLC** (high‐performance liquid chromatography) eluent was split to 100 µL min^−1^ just before the electrospray source. For analysis, the mass spectra were summed across the protein peak and the multiply charged ion envelope was deconvoluted into a molecular weight profile using the MaxEnt 1 module of MassLynx data analysis software (Waters).

### Panning of a Naïve LAC‐M library

A naïve llama, alpaca, camel phage library (LAC‐M)^[^ ^22^ ^]^ was first adsorbed onto subtraction wells coated with V_H_H FC5 and blocked with blocking buffer to remove/reduce the phage population binding to the plastic surface/blocking buffer. The pre‐adsorbed phages were then exposed to the target well(s) coated with the protein of interest (FC5‐DIE3IR). The pre‐absorption was a necessary step for panning of naïve libraries to ensure a specific enrichment for the protein target. The LAC‐M library was first phage‐rescued by growing 5 × 10^10^ cells in in 500 mL of 2YT‐tet (12.5 µg mL^−1^; Thermo Fisher Scientific, Waltham, Massachusetts) at 220 rpm, 30 °C overnight. The following morning, the culture cells were centrifuged at 5000 rpm, 4 °C for 15 min and the culture supernatant filtered through a 0.22 µm Stericup‐GP sterile vacuum filtration system (Sigma–Aldrich, St. Louis, Missouri). The phage supernatant was precipitated with 1/5 volume of PEG‐NaCl (500 mL + 100 mL; Sigma‐Aldrich, St. Louis, Missouri), incubated on ice for 1 h and then centrifuged at 10 000 rpm, 4 °C, for 15 min and the pellet was resuspended in 1 mL PBS. Bio‐panning started with coating of 40 µg FC5‐DIE3IR in PBS onto a single well of a NUNC MaxiSorp ELISA plate (Sigma–Aldrich, St. Louis, Missouri). One ELISA well was designated as a negative control coated with V_H_H FC5. The wells were sealed with parafilm and incubated overnight at 4 °C. At the same time, the phages rescued from the naïve LAC‐M library were pre‐incubated with StartingBlock (1:1 ratio; Thermo Fisher Scientific, Waltham, Massachusetts) and 20 µg of FC5 in a microcentrifuge tube and let to rotate gently overnight at 4 °C. The following morning, the wells were treated with 200 µL of StartingBlock at room temperature (**RT**) for 2 h. The pre‐incubated phage solution was spun down at 13 000 rpm, 4 °C for 10 min. The pre‐adsorbed phages were added to the negative control wells (V_H_H FC5) and incubated for 1 h at RT. The phage supernatant was transferred from the negative control well to the target antigen well (FC5‐DIE3IR) and incubated for an additional 1 h at RT. The supernatant containing the unbound phages was discarded and the well was rinsed five times with PBS‐0.1% (v/v) Tween 20 and then five times with PBS. The bound phages were eluted by adding 100 µL per well of 100 mm freshly prepared triethylamine. The contents of the well were then transferred into a microcentrifuge tube containing 50 µL 1 m Tris‐HCL, pH 7.4 and touch vortexed before being placed on ice. Exponentially growing TG1 cells were transfected with the eluted phages and incubated at 37 °C for 30 min. The TG1 culture was centrifuged and the pellet was resuspended in 1 mL 2YT‐Tet and the bacterial cells were spread on a large 2YT‐tet plate and incubated at 37 °C O/N. The following day, the cells were scraped off from the large plate using 5 mL 2YT‐Tet, rinsed with another 5 mL of 2YT‐Tet, and both cell mixtures were combined and grown at 220 rpm, 30 °C, 5 h. The culture was then centrifuged at 4100 rpm, 4 °C, for 30 min and the phage supernatant was precipitated with 1/5 volume of 20% PEG, 2.5 m NaCl on ice for 1 h and centrifuged at 4100 rpm, 30 min, at 4 °C. The phage pellet was resuspended in 200 µL StartingBlock and 100 µL of phage was used for a second round of panning. The panning was repeated for a total of four rounds as described above except that the washing with PBS‐T and PBS were increased to six, eight, and ten times, for rounds 2, 3 and 4, respectively. After four rounds of panning, 76 colonies were picked up from the dilution LB‐Tet plates and grown in 2 mL 2YT‐Tet overnight at 37 °C for a phage ‐ELISA experiment as described previously.^[^ ^22^ ^]^ The positive clones were colony‐PCR‐ed also as described previously.^[^ ^22^ ^]^ The PCR products of correct size were sequenced and the sequencing data were aligned to identify the unique V_H_H sequences. In total, six unique V_H_Hs with different CDR sequences were identified. The positive V_H_Hs were subcloned into pSJF2 expression vector and soluble V_H_Hs were expressed and purified by IMAC.^[^ ^22^ ^]^

### Generation of hNa_v_1.7‐HEK293 Cell Line

HEK‐293 cells (ATCC CRL1573) were transfected with human Na_v_1.7 plasmid (SC398916; OriGene Technologies, Rockville, MD, USA) as per Lipofectamine 2000′s protocol (Invitrogen Life Technologies P/N 52887). One day before transfection, HEK293 cells were seeded in 3 wells of a 6 well plate at a density of 8 × 10^5^ cells per well in growth medium (EMEM supplemented with 10% FBS). On the morning of a transfection, the growth medium was replaced and the DNA/Lipofectamine solution was prepared as follows: for each well, 9 µL of Lipofectamine 2000 was pre‐incubated with 125 µL of OptiMEM at room temperature for 5 min, then mixed with 125 µL of OptiMEME containing 3 µg of DNA. The DNA/Lipofectamine solution was then incubated for 20 min at room temperature prior to addition to cells. After 5 h of transfection, the medium was replaced. Two days post transfection, cells were combined, plated in 6 10 cm^2^ dishes, and grown in the presence 300 µg mL^−1^ G418 Sulfate, an antibiotic selective for transfected cells (400‐130‐IG, Wisent Bio Products, Saint‐Jean‐Baptiste, QC, Canada). Growth medium containing G418 Sulfate was replaced every 2 days. Visible colonies were selected and plated on cover slips for screening. Western Blot (**WB**) displayed a much higher level of hNa_v_1.7 protein in hNa_v_1.7‐HEK293 cells compared to non‐transfected cells (Figure S3, Supporting Information). Human Na_v_1.7‐HEK293 cells were further evaluated using patch‐clamp whole‐cell recordings to determine the functionality of the hNa_v_1.7 channels over‐expressed in the HEK293 cells (Figure S4, Supporting Information). When recorded using manual patch‐clamp whole‐cell technique, these cells showed functional channels with current amplitudes ranging between 2 and 10 nA (Figure S4, Supporting Information). Ten clones over‐expressing hNa_v_1.7 were expanded and frozen in liquid nitrogen for storage.

### Cell Preparation Protocols for Manual Patch‐Clamp Experiments

To prepare hNa_v_1.7‐HEK293 cells for manual patch‐clamp whole‐cell experiments, vials of frozen cells were thawed in a 37 °C water bath for ≈2 min or until completely thawed. Cells were then transferred to a 15 mL centrifuge tube and brought up to a volume of 10 mL with complete media (Eagles Minimum Essential Medium, **EMEM** + 10% Fetal Bovine Serum, **FBS** + G418). Cells were centrifuged at 1000 rpm for 5 min and the supernatant aspirated from pellet. The pellet was then re‐suspended in 6 mL of complete media. The pellet was gently triturated several times with a 5 mL pipette to break the cell pellet apart and remove clumps. The cell suspension was transferred to a T‐25 flask and maintained in a 37 °C/5% CO_2_ incubator and fed every 2 days with complete media. When cell confluency reached ≈70–80%, media was removed, cells washed 1x with 5 mL DPBS (no Ca^2+^, no Mg^2+^) and cells were then lifted with Cell Dissociation Solution (Sigma‐Aldrich, St. Louis, Missouri, USA; 3 min incubation at 37 °C) and split into T‐75 flasks at a 1:3 dilution. Cells were maintained in a 37 °C/5% CO_2_ incubator and fed every 2 days with complete media. Cells were split when confluency reached ≈70–80%. At the time of cell splitting, a dilute cell suspension was prepared in complete media (200 µL of cells plus 4.8 mL of media). 500 µL of this cell suspension was added to 10 wells of a 24‐well plate containing poly‐L‐lysine (0.025 mg mL^−1^; Sigma–Aldrich, St. Louis, Missouri, USA) coated 13 mm plastic coverslips (Thermanox Plastic coverslips, Thermo Fisher, Waltham, Massachusetts, USA). The coverslips were kept in the incubator for 24–48 h before being used for experiments.

Cryopreserved rat DRGs were kindly provided by QBM Cell Science (Ottawa, ON, Canada). A vial of frozen DRGs (1 × 10^5^ cells/0.25 mL) was thawed in a 37 °C water bath. Cells were then transferred to a 15 mL centrifuge tube. To avoid osmotic shock, pre‐warmed media (Neurobasal + 1x B‐27, Thermo Fisher, Waltham, Massachusetts, USA) was added dropwise onto cells over a period of ≈2 min while rotating tube by hand. Cell suspension was mixed 1x by careful inversion of the 15 mL tube. Cells were plated onto 13 mm plastic coverslips (Thermanox Plastic coverslips, Thermo Fisher, Waltham, Massachusetts, USA) coated with poly‐d‐lysine (0.1 mg mL^−1^; Sigma–Aldrich, St. Louis, Missouri, USA). Four h after plating the media (Neurobasal + B27) was replaced with Neurobasal + B27 media containing 0.0875 µg mL^−1^ Uridine (UDR; Sigma–Aldrich, St. Louis, Missouri, USA) and 0.0375 µg mL^−1^ 5‐fluoro‐2‐deopxyuridine (FUDR; Sigma–Aldrich, St. Louis, Missouri, USA). Cells were incubated at 37 °C and 5% CO_2_ until time of use.

### Manual Patch‐Clamp Whole‐Cell Experiments

Whole‐cell patch‐clamp recordings were obtained with a Multiclamp 700B amplifier (Molecular Devices, San Jose, CA, USA) controlled with a pClamp (v11.2) software (Molecular Devices, San Jose, CA, USA) used in combination with a Digidata 1440A A/D converter (Molecular Devices, San Jose, CA, USA). Data were acquired at 20 kHz and filtered at 2 kHz.

For voltage‐clamp recordings, borosilicate pipettes had a resistance of < 2 MΩ when filled with a solution containing (mm): CsF, 140; NaCl, 10; MgCl_2_,1; HEPES, 10; ATP‐Mg^2+^, 2; GTP‐Mg^2+^, 0.2. The pH was adjusted to 7.3 with CsOH. Pipettes were pulled from borosilicate glass using a P‐97 Flaming‐Brown type micropipette puller (Sutter Instrument, Novato, CA, USA) and fire‐polished with a microforge (MF‐830; Narishige, Japan). To record Na^+^ currents, the cells were maintained in an extracellular solution containing (in mm): NaCl, 150; CsCl, 3; CaCl_2_, 1; MgCl_2_, 1; HEPES, 10. The pH was adjusted to 7.3 with NaOH. The cells were kept at a holding potential of −60 mV. Series resistance (**R_s_**) was compensated at 70%. The stimulus protocol to evoke Na^+^ currents consisted of 1 Hz frequency, and 20 ms duration voltage steps elicited from a conditioning pre‐pulse at −120 mV to a potential that evoked maximal inward Na^+^ current. The test potential was determined by stimulating the cell to various potential levels around the peak activation potential of hNa_v_1.7 (from −40 to −20 mV). The stimulus potential that generated the greatest magnitude of inward current was used for subsequent experimental protocols. The stimulus protocol was designed to hold hNa_v_1.7 channels in a resting, non‐inactivated state. Cells were allowed to stabilize for up to 5 min following initial breaking of the membrane into the whole‐cell configuration. Following observation of a stable baseline, the experimental protocol was initiated. Two‐minute baseline recordings were followed by recordings during V_H_H exposure. V_H_Hs were applied through static bath application. The effect of the V_H_Hs was assessed comparing the amplitudes of the currents in control and in the presence of the V_H_H.

For current‐clamp recordings of DRGs, borosilicate pipettes had a resistance of 2–2.5 MΩ when filled with a solution containing (mm): K^+^‐gluconate, 130; HEPES, 10; KCl, 10; K^+^‐ATP, 2; Mg^2+^‐ATP, 2; Na_2_GTP, 0.5; pH = 7.3; 280 mOsmol. To record action potential firing, the DRGs were maintained in an extracellular solution containing (in mm): NaCl, 133; KCl, 4; CaCl_2_, 2; MgCl_2_, 1; Glucose, 5; HEPES, 10. Osmolarity was 280 mOsm. The pH was adjusted to 7.4 with NaOH. The cells were kept at their resting potential and action potentials were evoked by injecting 500 ms current steps, ranging from −80 to +90 pA with 2–5 pA incremental steps unless otherwise indicated. Electro‐responsive properties were also measured. Values of amplitude, threshold and overshoot of action potential (AP) were measured on the first elicited AP. The threshold of AP was identified by superimposing the first AP evoked in each cell in control and in presence of V_H_H DI‐D and measuring the potential at which the rising phase of AP starts.

Cryopreserved rat DRGs (QBM Cell Science, Ottawa, ON, Canada) and cryopreserved neurons in general were known to have really high input resistance (**R_in_**). DRG neurons had an average R_in_ of 1273 ± 195 MΩ (n = 8) were recorded.

Stock solutions of each V_H_H were kept at −80 °C to avoid degradation. Before the experiment the stocks were diluted 20 times in extracellular recording solution. V_H_H solutions were prepared in a manner allowing addition of 50 µL of solution to the recording chamber containing the cells. The 50 µL of V_H_H solution was statically added to 650 µL of extracellular recording solution present in the recording chamber.

Analyses were performed off‐line with the software, IGOR (WaveMetrics Inc., Portland, Oregon, USA) and/or Origin 2017 (OriginLab Corporation, Northampton, MA, USA). Statistical significance of the results was determined with two‐tailed Student's *t*‐tests. All values were expressed as means ± SEM, and a *p*‐value of <0.05 was considered significant.

### Cell Preparation Protocol for High‐Throughput Automated Patch‐Clamp Experiments Using SyncroPatch 384PE

Four engineered cell lines were used for SyncroPatch experiments: hNa_v_1.7‐HEK293 cell, hNa_v_1.1‐HEK293 cells, hNa_v_1.2‐CHO cells (Catalog Number CT4010; ChanTest, Cleveland, OH, USA) and hNa_v_1.5‐HEK293 cells (Catalog Number CT6207; ChanTest, Cleveland, OH, USA).

HNa_v_1.7‐HEK293growth medium consisted of EMEM, 10% FBS and 300 µg mL^−1^ G418. All medium components came from Wisent (St. Bruno, QC, Canada). HNa_v_1.1‐HEK293 cells growth medium consisted of DMEM /High Glucose (Cytiva, Marlborough, Massachusetts, USA), 10% FBS, 1 mg mL^−1^ G418, 1x Penicillin Streptomycin, 3 µg mL^−1^ Puromycin (Sigma–Aldrich, St. Louis, Missouri, USA). FBS, G418 and Pen/Strep purchased from Wisent (St. Bruno, QC, Canada). HNa_v_1.5‐HEK293 cells (Catalog Number CT6207; ChanTest, Cleveland, OH, USA) growth media consisted of DMEM/F12, 10% FBS, 0.5 mg mL^−1^ G418 and 1x Pen/Strep (Wisent, St. Bruno, QC, Canada). HNa_v_1.2‐CHO EZ cells (Catalog Number CT4010; ChanTest, Cleveland, OH, USA) growth media consisted of DMEM/F12, 10% FBS (Wisent, St. Bruno, QC, Canada).

HNa_v_1.7‐HEK293 cells, hNa_v_1.1‐HEK293 cells and hNa_v_1.5‐HEK293 cells, were cultured in T‐75 flasks at 37 °C, 5% CO_2_ and split when confluency reached 70–80% to retain surface channel expression. Cells were harvested for use when confluency reached ≈70%. To harvest cells, the media was aspirated and cells were washed 2x by the addition and aspiration of 5 mL of **DPBS** (Dulbecco's Phosphate Buffered Saline, no Ca^2+^, no Mg^2+^; Gibco, Thermo Fisher, Waltham, Massachusetts, USA). Post washing, 2.5 mL of Accutase (Sigma–Aldrich Corporation, Saint Louis, USA) was added to flasks which were then rocked to ensure complete coverage.  Flasks were incubated at 37 °C in a 5% CO_2_ incubator for 15 min. Flasks were gently rocked to dislodge the cells and 8 mL of serum free EMEM added to flasks to dilute the Accutase and inhibit cell digestion. Cells were gently triturated with a 5 mL pipette and then transferred to a 15 mL centrifuge tube. Cells were centrifuged at 1000 rpm for 4 min, and re‐suspended in chilled (4 °C) External Solution (Nanion Technologies, Munich, Germany) at a concentration of 1–5 × 10^6^ cells mL^−1^.

HNa_v_1.2‐CHO EZ cells were thawed and plated into 100 mm tissue culture dishes. Cells were incubated at 37 °C in a 5% CO_2_ incubator for 2 h prior to harvesting. Harvesting was the same as described above.

The patch‐clamp whole‐cell experiments using a SyncroPatch 384PE (Nanion Technologies, Munich, Germany) were performed with the following solutions: i) internal solution for cell recording contained (in mm) CsF, 110; NaCl, 10; CsCl, 10; EGTA,10; Hepes,10 (285 mOsm; pH adjusted to 7.2 with 1 N CsOH); ii) external solution contained (in mm) NaCl, 140; KCl, 4; CaCl_2_, 2; MgCl_2_, 1; glucose, 5; HEPES, 10 (298 mOsm; pH adjusted to 7.4 with 1 N NaOH). All solutions were purchased from Nanion Technologies, Munich, Germany.

### Electrophysiological Protocols

The current–voltage (**I–V**) relationship was obtained by plotting the amplitudes of the evoked inward currents in response to voltage steps versus the potentials of the steps (voltage steps from −80 to +40 mV in 10 mV increments) following a 100 ms conditioning pre‐pulse at −120 mV. In the experiments on Na_v_1.2, the duration of the pre‐pulse was lengthened to 200 ms, as per the cells supplier's instructions (ChanTest, Cleveland, OH, USA). The activation was calculated by plotting the normalized peak Na^+^ current conductance (G_Na_) versus the voltage evoking the current and fitting the resulting curve to a Boltzmann equation. The conductance G_Na_ was calculated using the Equation:

(1) $$G_{\mathrm{Na}}=I_{\mathrm{Na}}{\left(V-V_{\mathrm{rev}}\right)}^{-1}$$

where I_Na_ is the peak current evoked by the test depolarizing pulse (V), and V_rev_ is the Na^+^ reversal potential. Steady‐state fast inactivation was calculated by exposing the cells to a series of conditioning pre‐pulses at voltages ranging from −150 to +20 mV for 200 ms and then subjecting the cells to a test pulse at 0 mV for 25 ms. Normalized residual peak currents were plotted versus pre‐pulse potentials and curves were fitted to the Boltzmann equation. To measure the steady‐state slow inactivation, cells were exposed to a series of conditioning pre‐pulses at voltages between −120 and +80 mV of a duration of 2 s. The conditioning pre‐pulses was followed by a 100 ms period at −120 mV and by the test stimulus at 0 mV of a duration of 40 ms. Normalized residual peak currents were plotted versus pre‐pulse potentials and curves fitted to the Boltzmann equation. The deactivation protocol consisted of a conditioning period at −120 mV followed by a 1 ms voltage step to −10 mV and a series of 50 ms test pulses from −120 to −50 mV with 10 mV increments to elicit Na_v_1.7 tail currents. The decay time (from peak to 20% peak amplitude) of tail current decay was then plotted as a function of the test pulse. The time to peak was measured in the current‐voltage relationship as the time that spanned between the beginning of the voltage pulse and the moment the evoked current reached the peak. This value was plotted as a function of the amplitude of the voltage pulse. The inactivation decay time was measured in the current–voltage relationship as the time necessary for the current to decrease from the peak to 20% of peak amplitude. This time was then plotted versus the voltage of the pulse evoking the current.

In SyncroPatch experiments Rs was not compensated due to high likelihood of oscillations caused by the system. The average value of the Rs was 17.5 ± 0.2 MΩ in control (n = 112) and 16.7 ± 0.2 MΩ after V_H_H DI‐D application (n = 137). The average **R_seal_** (seal resistance) was 1.13 ± 0.01 GΩ in control and 1.21 ± 0.02 GΩ with the V_H_H DI‐D (R_s_/R_seal_ of 0.015 and 0.011, respectively).

### Analysis of SyncroPatch 384PE Data

Only the wells (cells) in which the seal resistance was higher than 500 MΩ and the peak current (measured at −10 mV in the *I*–*V*) comprised between −800 pA and −5 nA, were analyzed. Analyses were performed off‐line with the software DataControl (Nanion Technologies, Munich, Germany), IGOR (WaveMetrics Inc., Portland, Oregon, USA) and/or Origin 2017 (OriginLab Corporation, Northampton, MA, USA). Statistical significance of the results was determined with two‐tailed Student's *t*‐tests. All values were expressed as means ± SEM, and a *p*‐value of < 0.05 was considered significant.

### Simulation of Na_v_1.7 Currents

Na_v_1.7 currents were simulated by numerically integrating the differential equations for the occupation probabilities of the different states in a simplified kinetic scheme^[^ ^50^ ^]^ using the Euler method in an Excel spreadsheet. The values of the transition rate constant at four different voltages (−30, −10, 0, and +20 mV) were changed individually in order to minimize the difference between the measured and the simulated current. Their voltage dependence was then assessed by curve fitting these values to a single exponential function.

### Surface Plasmon Resonance (SPR)

Control V_H_H FC5 and human‐, rat‐ mouse‐ FC5‐DIE3IR proteins were SEC purified using a Superdex 75 column (Cytiva, Vancouver, Canada) connected to an AKTAPurifier (Cytiva) in HBS‐EP+ running buffer (10 mm HEPES, pH 7.4, containing 150 mm NaCl, 3 mm EDTA and 0.05% (v/v) surfactant P20) at a flow rate of 1 mL min^−1^. For SPR analysis, a Biacore T200 instrument (Cytiva) was used with HBS‐EP+ running buffer at 25 °C. FC5‐DIE3IR and FC5 proteins were amine coupled (300–400 response units, RU) on series S CM5 sensorchips in 10 mm acetate buffer, pH 4.0. An ethanolamine blocked flow cell served as a reference surface. Using single cycle kinetics, monomer fractions of SEC‐purified V_H_H DI‐D were flowed over the amine coupled FC5‐DIE3IR and FC5 surfaces at concentrations ranging from 312 nm to 5 µm at a flow rate of 20 µL min^−1^, with 60 s of contact time and 180 s of dissociation time. Equilibrium dissociation constants (*K*_D_) were determined by steady state analysis from reference flow cell‐subtracted sensorgrams using the Biacore T200 BIAevaluation software v3.0. Surfaces were regenerated with HBS‐EP+ running buffer.

### Hydrogen‐Deuterium Exchange Mass Spectrometry (HDX‐MS)

FC5‐DIE3IR and purified V_H_H DI‐D were diluted to 40 and 20 µm, respectively, and mixed at a 1:1 ratio and kept at 25 °C. For the unbound control, FC5‐DIE3IR was diluted to 20 µm with PBS. Labelling reactions were initiated by adding 3 µL of IGF1R complexes to 3 µL of 90% D_2_O at 25 °C. Labelling was quenched after 1, 10, and 60 min by the addition of 39 µL ice‐cold 8 m urea, 500 mm TCEP, 200 mm Gly‐HCl, pH 3.0, and samples were allowed to reduce for 2 min on ice. The sample was loaded into a 20 µL loop and manually injected in a custom valve cooler. This was followed by digestion at room temperature with a Poroszyme Immobilized Pepsin Cartridge (2.1 × 30 mm, Thermo Scientific) at 50 µL min^−1^ for 1.5 min, followed by washing/trapping onto a C18 PepMap100 cartridge kept at 1 °C (5 µm, 1 × 5 mm, Thermo Scientific) at 400 µL min^−1^ for 1 min in mobile phase A (0.23% formic acid in water). Peptides were eluted at 1 °C from a Biobasic‐18 (5 µm, 0.32 × 50 mm, Thermo Scientific) analytical column at 10 µL min^−1^ with a 10–35% mobile phase B gradient (0.23% formic acid in acetonitrile) over 6 min. LC‐MS analysis was performed with an Agilent 1260 Infinity pump coupled to a Q‐Tof Ultima API mass spectrometer (Water). Peptides were identified with Mascot and validated using an unlabeled sample injection. Data was collected in triplicate and analyzed with MS studio, and mass shifts were considered significant if greater than >2 SD with a *p*‐value of 0.05.^[^ ^76^ ^]^

### In Vivo Animal Experiments

For in vivo experiments, rats and mice were maintained at the National Research Council Canada (NRC) in accordance with the guidelines of the Canadian Council on Animal Care. All procedures performed on animals in this study were in accordance with regulations and guidelines reviewed and approved by the NRC Animal Care Committee under AUP2022.06 (Hargreaves model of hyperalgesia, approved on June 13th 2022), AUP2020.17 (OD1 mouse pain model, approved on January 21, 2021), AUP 2020.09 (Neuropathic pain model, approved on July 30, 2020), AUP2023.04 (Formalin pain model, approved on May 6, 2023) and AUP2018.04 (Pharmacokinetics studies, approved on May 16, 2018).

Rats aged between 4 and 6 weeks (weight range, 180–220 gr) were used for intraplantar (**ipl**) administrations of various antibodies and evaluation of their efficacy in the Hargreaves model of inflammatory pain. Inflammatory pain was induced by injecting a low volume (50 µL) of complete Freund's adjuvant (CFA; heat‐killed M. Tuberculosis; Sigma, St. Louis, MO) suspended in oil:saline 2:1 emulsion, into the right hind paw. After 48 h of the CFA injection, the baseline for latency paw withdrawal was measured and test compounds were injected under light isoflurane anesthesia. The paw withdrawal latency in response to the application of a radiant stimulus onto the plantar surface of both right and left paw was measured using the plantar Analgesia Meter equipment for paw stimulation (IITC Life Science, Woodland Hills, CA) for up to 4 h post injection. The time taken by the animal to respond by licking or flicking its paw was interpreted as a positive response (paw withdrawal latency). A cut‐off time (20 s) was established at the end of which the heat source shut off automatically to avoid tissue damage. Animals were kept (randomized) one per cage and staff performing pain experiments were blinded to the content of injectable compounds. The V_H_H effect was compared with the effect of the small molecule TC‐N1752 (Tocris, Bristol, United Kingdom) and negative control V_H_H A20.1. TC‐N1752 has IC_50_ values of 0.17, 0.3, 0.4, and 1.1 µm for hNa_v_1.7, hNa_v_1.3, hNa_v_1.4, and hNa_v_1.5, respectively. Percentage of maximum possible effect (%MPE) was calculated using the formula %MPE = (Test Latency – Baseline Latency) x 100/(Cut‐off – Baseline Latency). Area under the curve (AUC) from %MPE graph was calculated by the trapezoidal method.

The OD1 model was performed as previously described by Deuis and collaborators^[^ ^21^ ^]^ with minor modifications. Briefly, CD‐1 mice aged between 4 and 6 weeks (weight range, 25–30 g) were kept under 12 h light‐dark cycles and received standard rodent chow and water ad libitum. All animals were allowed to acclimatize to the facility, staff and equipment prior to the beginning of experiments. For the first 2 weeks mice were placed in polyvinyl boxes (10 × 10 × 10 cm) for acclimatization to the experimental setup. Approximately ten sessions of 30 min each were required. On the day of experiment, test compounds were injected subcutaneously into the dorsal side of the left hind paw 30 or 60 min before OD1 injection. OD1 (Tocris Bioscience, Bristol, United Kingdom) was freshly diluted in PBS and administered by shallow subcutaneous injection into the dorsal side of the hind paw of the mouse. The volume of all subcutaneous injections was 30 µL and the procedure was carried out with animals under light isoflurane anesthesia. Mice were then placed into polyvinyl boxes with a camera mounted underneath for 20 min video recordings. Spontaneous pain behavior (licks and flinches) following OD1 injection were later analyzed by a blinded investigator in 5 min bin times.

Intrathecal delivery of test compounds was performed by lumbar punctures into the L5–L6 intervertebral space of mice under isoflurane anesthesia using a 50 µL Hamilton syringe connected to a 30‐gauge needle as previously described.^[^ ^77^ ^]^ A successful puncture of the dura was indicated by a reflexive lateral flick of the tail or formation of an “S” shape by the tail. The volume of each drug was 5 µL. The experiments were performed only after each administrator had achieved a success rate of >90% in intrathecal injection training sessions, which involved the administration of NMDA (3 pmol in 5 µL).

A modified formalin pain model was used as previously described.^[^ ^48^ ^]^ Briefly, 30 min before formalin injection, CD‐1 male mice (4–6 week‐old; weight range, 25–30 g) were intrathecally injected with the test compounds and placed in their home cages without any bedding or environmental enrichment items. Formalin (30 µL at 2.5% concentration of formaldehyde diluted with phosphate buffered saline; Sigma, St. Louis, MO, USA) was injected subcutaneously using a syringe with a 30‐Gauge needle into the right hind paw of mice anesthetized with light isoflurane. Pain behavior (licking, lifting, shaking and biting of the injected paw) was scored for 60 min post injection by a rater that was blind to the experiments. The time course and the area under the curve (AUC) graphs were calculated.

Spared nerve injury (SNI) model was induced as previously described.^[^ ^49^ ^]^ Briefly, male Sprague Dawley rats weighing 200–250 g were placed under isoflurane anesthesia and the right thigh shaved. The skin and the underlying biceps femoris muscle were incised and the sciatic nerve at its trifurcation point was exposed. The tibial and common peroneal nerves were ligated and then severed, leaving the sural nerve intact. The wounds were then closed and the rats were allowed to recover prior to injection of test compounds.

All SNI rats were tested for mechanical allodynia 1 day before surgery, 7 days after surgery and then at 1, 2, 4, and 24 h post intrathecal injection of test compounds. Mechanical allodynia was assessed with a dynamic plantar aesthesiometer (Ugo Basile, Comerio, Italy), which has an automated von Frey‐type filament (0.5 mm diameter). To measure mechanical thresholds of the hind paw, rats were placed in a plastic cage with a wire mesh floor and allowed to acclimate for 30 min before each test session. A paw‐flick response was elicited by applying an increasing force (measured in grams, g) directed to the plantar surface of the hind paw. The force applied was initially below the detection threshold, then increased from 1 to 50 g in 0.5 g steps over 20 s. The rate of force increase was 2.5 g s^−1^. The force to elicit a reflex removal of the hind paw was monitored. This was defined as the mean of three measurements made at 5 min intervals.

### Biodistribution and Pharmacokinetic Study

For the biodistribution studies, succinimidyl ester (NHS ester) linked CF770 (Biotium Inc., Fremont, CA, USA) was conjugated to either V_H_H DI‐D or V_H_H A20.1 using methods recommended by the manufacturer. Briefly, CF770‐NHSester powder was solubilized in DMSO to create a 31.38 mg mL^−1^ solution. A 6‐fold molar excess of CF770‐NHSester in 10% (v/v) carbonate buffer (pH 9.3) was added to either V_H_H DI‐D or V_H_H A20.1 in PBS (pH 7.4), in a tube while mixing. The reaction was then incubated at room temperature for 1 h with slow mixing. Unreacted dye was removed using an Amicon Ultra‐4 Centrifugal Filter Unit with a 10k cutoff membrane and the labeled V_H_H DI‐D or V_H_H A20.1 were re‐suspended in PBS, pH 7.4.

V_H_H DI‐D labeled with CF770 (V_H_H DI‐D‐CF770) or V_H_H A20.1 labeled with CF770 (V_H_H A20‐CF770) were injected via the tail vein in CD‐1 mice at a dose of 0.4 mg kg^−1^. In vivo optical imaging studies were performed using an IVIS Lumina III preclinical animal imager (Perkin Elmer, Waltham, Massachusetts, USA). Animals were imaged at pre‐scan, 10 min, 30 min, 1 h, 2 h, 4 h, 6 h and 24 h in both dorsal and ventral positions. At the end of the experiment (6 and 24 h), animals were perfused with heparinized saline, and dissected organs (brain, heart, lung, liver, spleen, kidney, spine, thyroid, skeletal muscle, testes) and lymph nodes (mandibular, axial/brachial, inguinal, mesenteric, and popliteal) were imaged ex vivo. Total fluorescence radiance efficiency was determined from whole organ region of interest (ROI) using the Living Image 4.1 software (Perkin Elmer, Waltham, Massachusetts, USA).

For pharmacodynamic study, male Wistar rats (n = 3/group, body weight = 352.3 ± 10.2; Charles River Laboratories, Hollister, CA, USA) were intravenously (*iv*) injected via tail vein with 4 mg kg^−1^ of V_H_H DI‐D. Serial blood samples (≈200 mL) were collected at pre‐dose and at 5 min, 10 min, 15 min, 30 min, 1 h, 2 h, 4 h, 6 h, and 24 h post‐injection. To separate the serum, blood was allowed to clot at room temperature (RT) for 30 min and then centrifuged at 1500 G for 10 min at RT. Following centrifugation, the supernatant was transferred into the pre‐labelled tubes, snap‐frozen immediately on dry ice and stored at −80 °C.

## Conflict of Interest

The authors declare no conflict of interest.

## Supporting information

Supporting Information

## Data Availability

The data that support the findings of this study are available from the corresponding author upon reasonable request.
