# Supplementary material for: A Novel Antigen Design Strategy to Isolate Single‐Domain Antibodies that Target Human Nav1.7 and Reduce Pain in Animal Models
Source: Adv Sci (Weinh). 2024 Aug 29;11(40):2405432. doi: 10.1002/advs.202405432 (PMC11516162; doi:10.1002/advs.202405432)
Supplement: Supplementary file 1 — Supporting Information [file ADVS-11-2405432-s001.docx]

Supporting Information

A Novel Antigen Design Strategy to Isolate Single-domain Antibodies that Target Human Nav1.7 and Reduce Pain in Animal Models

Marzia Martina*, Umberto Banderali, Alvaro Yogi, Mehdi Arbabi-Ghahroudi, Hong Liu, Traian Sulea, Yves Durocher, Greg Hussack, Henk van Faassen, Balu Chakravarty, Qing Yan Liu, Umar Iqbal, Binbing Ling, Etienne Lessard, Joey Sheff, Anna Robotham, Debbie Callaghan, Maria Moreno, Tanya Comas, Dao Ly and Danica Stanimirovic

**
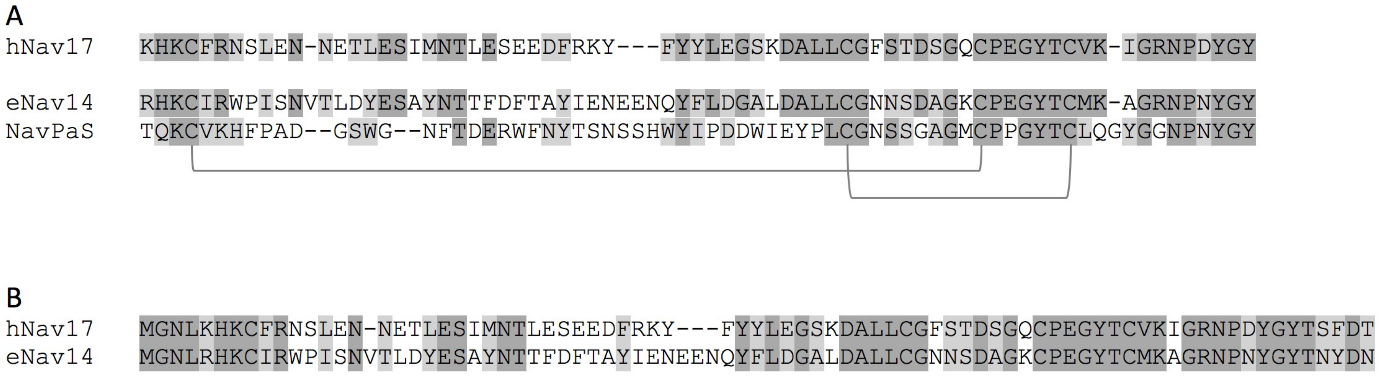
**

**Figure S1.** Sequence alignments between the DIE3IR loop of hNa_v_1.7 and homologous eukaryotic sodium channels with 3D structures available at the time of the immunogen design. (A) Alignment to electric eel Na_v_1.4 and American cockroach Na_v_PaS. Disulfide connectivity is indicated. (B) Alignment to electric eel Na_v_1.4 used for homology modeling. Dark and light gray shading highlight identical and homologous amino-acid residues, respectively.

**
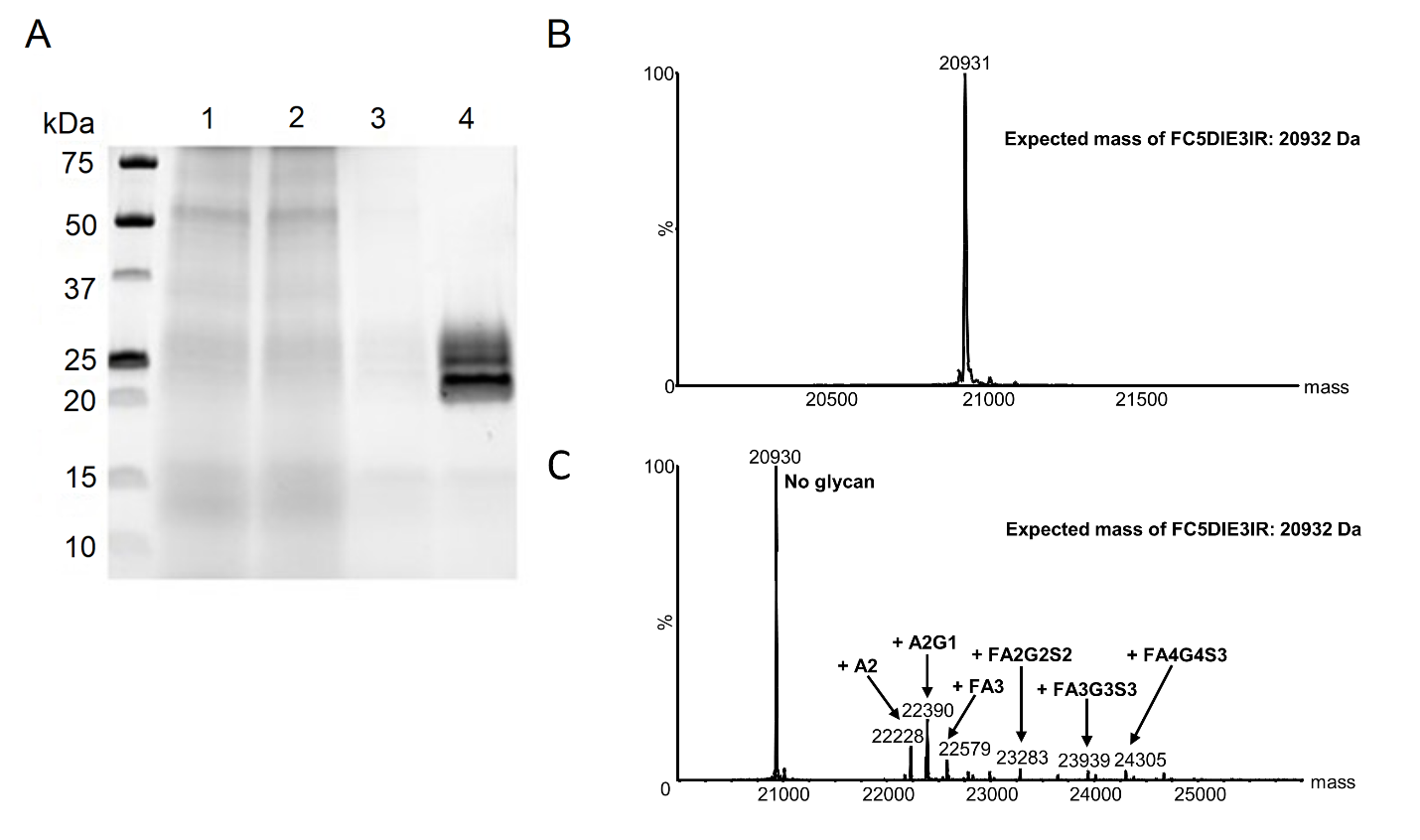
**

**Figure S2**. (A) SDS-PAGE gel (reducing conditions). Recombinant protein FC5-DIE3IR produced in CHO-3E7 cell line. The lanes on the gel represent, 1, Harvest 11 days post-transfection (25 µL); 2, flow-through (FT) on MabSelect Protein A column (25 µL); 3, PBS wash (25 µL); and 4, FC5-DIE3IR product desalted (5 µg). Note that purified protein migrates as a smear on the gel due to possessing one N-glycosylation site. (B) Intact mass LC-MS of 5 µg FC5-DIE3IR after overnight 37°C treatment with PNGaseF. (C) Intact mass LC-MS of 5 µg FC5-DIE3IR (no PNGaseF treatment). FC5-DIE3IR glycoforms labelled with Oxford naming.

**
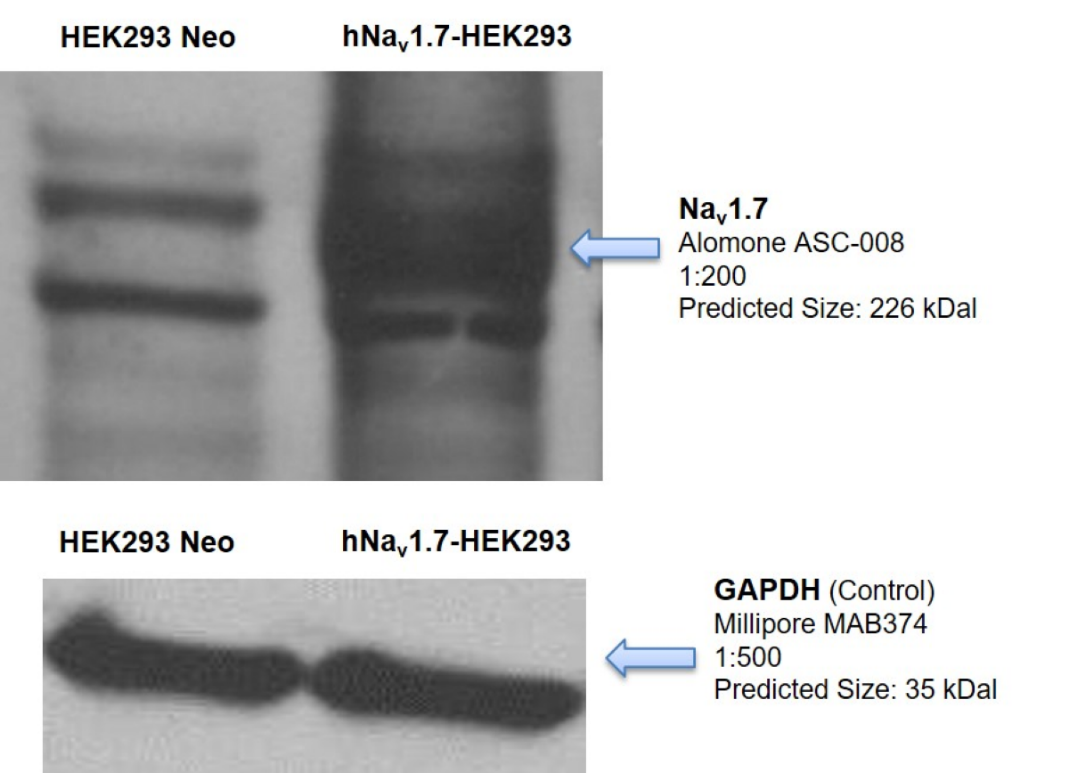
**

**Figure S3**. Western Blot. WB experiment to examine levels of Na_v_1.7 protein in hNa_v_1.7-HEK 293 cells and HEK293 Neo (non-transfected) cells lysate (10 µg of proteins were loaded).

**
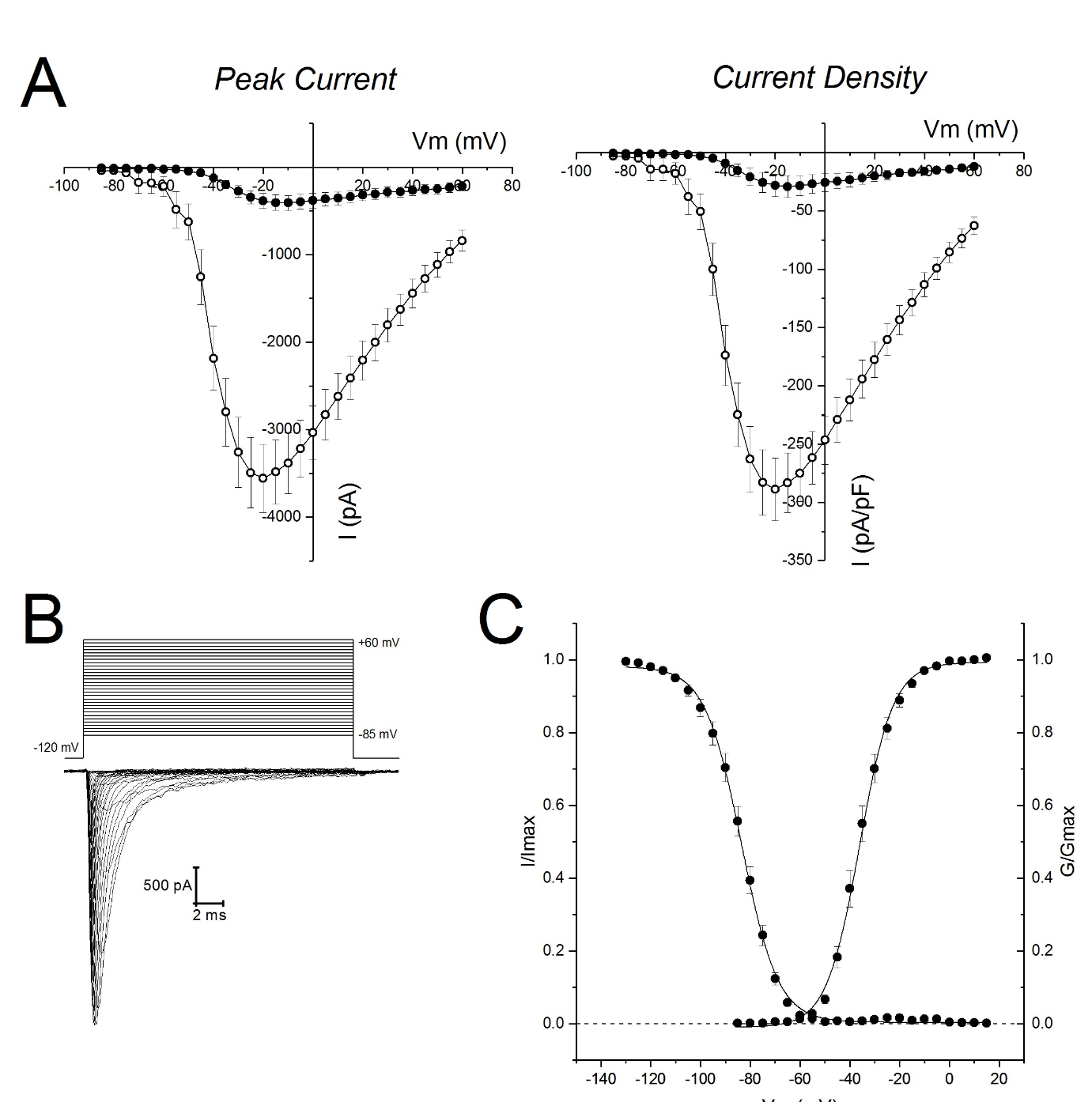
**

**Figure S4.** Human Na_v_1.7 currents in HEK293 cells stably transfected with the SCN9A plasmid. HEK293 cells stably transfected with the SCN9A plasmid show significantly larger Na^+^ currents (hNa_v_1.7-HEK293; n = 41) compared to non-transfected HEK293 cells (Control HEK293 cells; n = 13). (A) Current-voltage relationship showing peak current amplitude (left panel) and current density (right panel) in hNa_v_1.7-HEK293 cells (open circles) as compared to non-transfected HEK293 cells (filled circles). (B) Raw traces of family of sodium current (lower trace) generated from a series of voltage steps from -85 to +60 mV in 5 mV increments (upper trace). (C) Activation and inactivation traces from hNa_v_1.7-HEK293 cells. Half-maximal activation occurred at -35.99 ± 0.29 mV (n = 20). Half-maximal inactivation occurred at -83.45 ± 0.22 mV (n = 19). Statistics shown as mean ± SEM.

**

**

**Figure S5**. Effect of the application of DI-A, DI-B, DI-C and DI-H on the kinetics of the Na_v_1.7 currents recorded using SyncroPatch 384PE in HEK293 cells overexpressing hNa_v_1.7 channels. (A) Current-voltage (I-V) relationships showing peak current amplitude in control (full square; n = 51) and in the presence of DI-A (empty circles; n = 57), DI-B (empty upward triangle; n = 45), DI-C (empty downward triangle; n = 55) and DI-H (empty diamond; n = 55). (B) Activation and fast inactivation traces. (C) Steady state slow inactivation. (D) Voltage dependence of the deactivation currents decay. (E) Voltage dependence of time to peak. (F) Voltage dependence of inactivation time constant. Statistics shown as mean ± SEM.

**
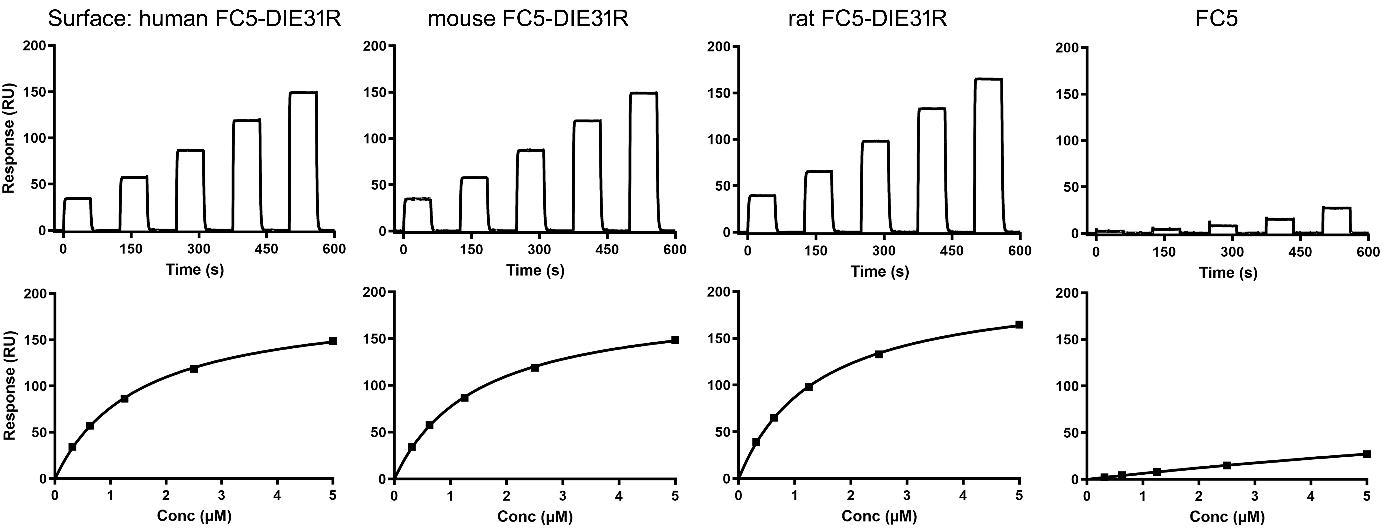
**

**Figure S6.** SPR sensorgrams and steady state plots demonstrating the specific interaction of V_H_H DI-D and FC5-DIE3IR from various species. SEC-purified monomeric V_H_H DI-D (0.3 – 5 µm) was flowed over amine coupled FC5-DIE3IR surfaces. Raw SPR sensorgram binding data (*top*) and steady state affinity plots (*bottom*) were used to determine the equilibrium dissociation constants (*K*_D_s; Table S3, Supporting Information).

**
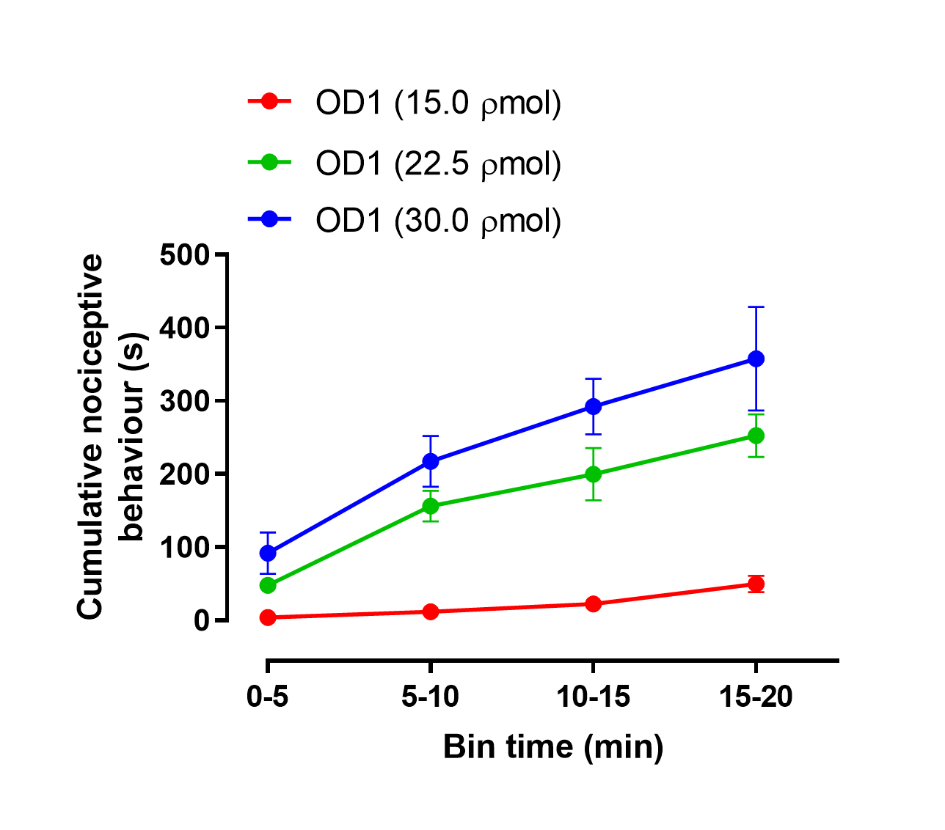
**

**Figure S7.** OD1-induced mouse model of Na_v_1.7-mediated pain. Intraplantar injection of OD1 (15-30 pmol of OD1 in 30 µL) induces spontaneous pain behaviors, as evidenced by licking, flinching, lifting and shaking of the injected hind paw. Spontaneous pain was quantified on a 20 min period in 5 min intervals. Data are shown as mean ± SEM of 3 mice per group.

**
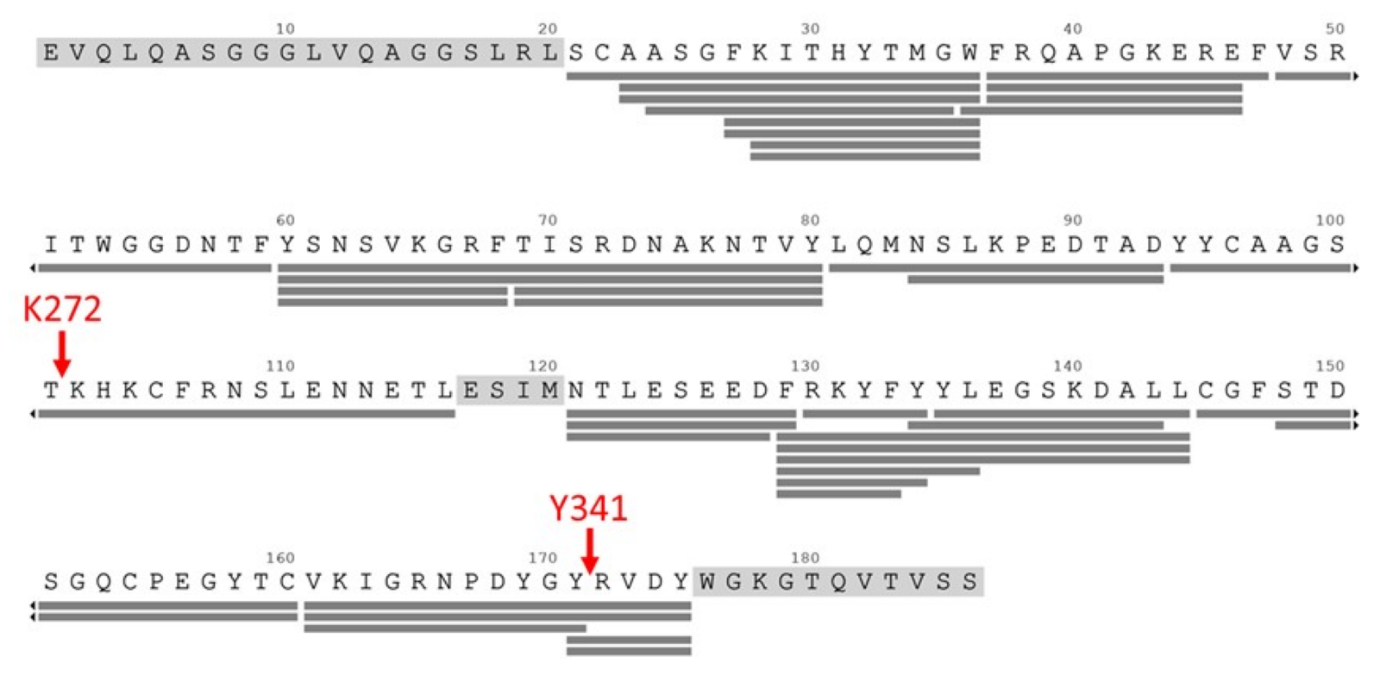
**

**Figure S8**. Validated sequence coverage map. Each rectangle represents a peptide for which deuteration was measured across all three time points. Red arrows indicate insertion of 70-aa DIE3IR peptide; native Na_v_1.7 numbering is shown. A redundancy of 2.3 was achieved, with 41 peptides covering 81% of the sequence. Coverage maps were plotted with MS Studio.

**
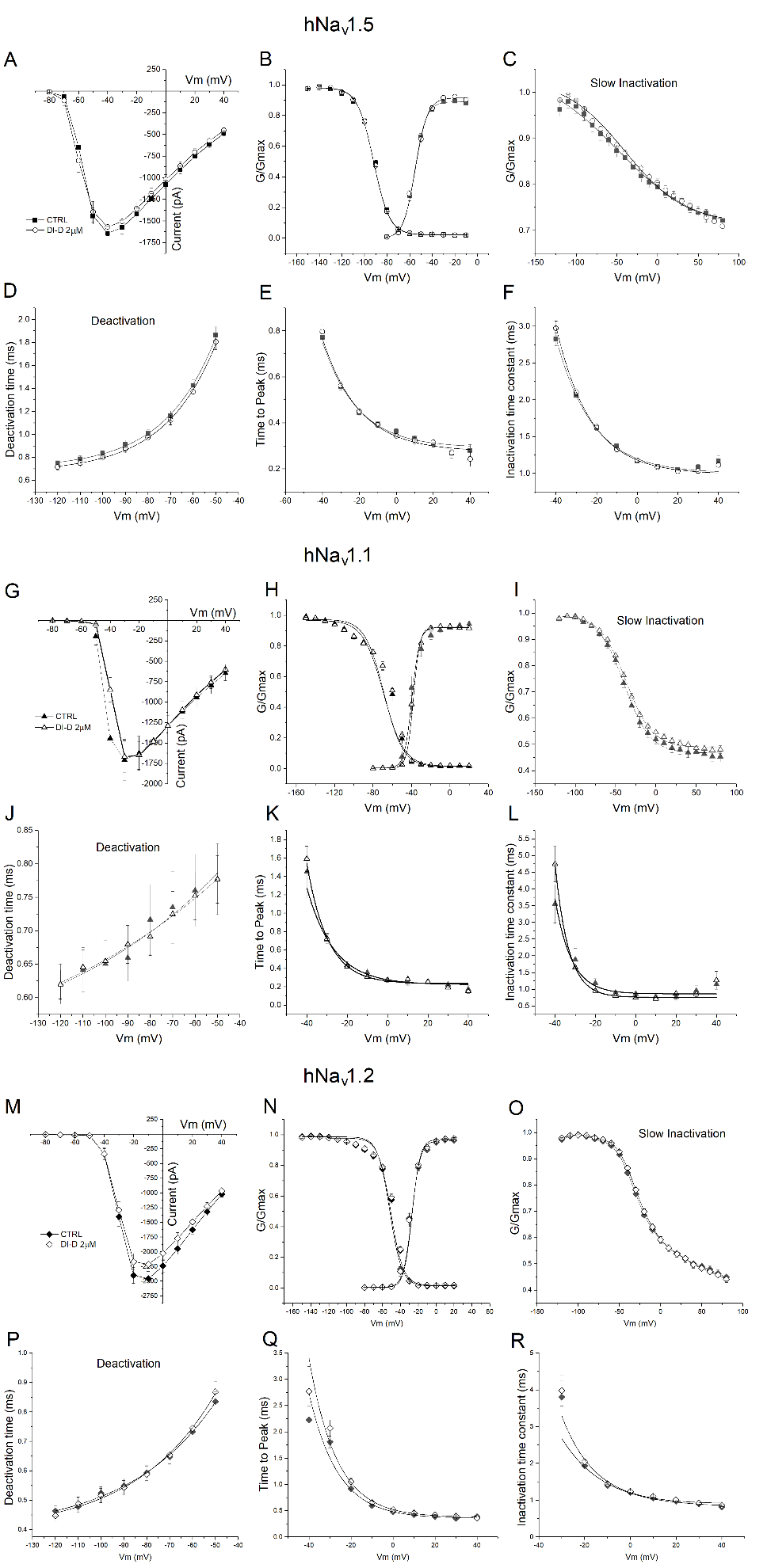
**

**Figure S9**. Effect of the application of V_H_H DI-D on the kinetics of the hNa_v_1.1, hNa_v_1.2 and hNa_v_1.5 currents recorded using SyncroPatch 384PE in HEK293-hNa_v_1.1, CHO-hNa_v_1.2 and HEK293-hNa_v_1.5 channels. hNa_v_1.5 currents: (A) Current-voltage (I-V) relationship showing peak current amplitude in control (CTRL, full square; n = 93) and in the presence of V_H_H DI-D (empty circles; n = 90). (B) Activation and fast inactivation traces. (C) Steady state slow inactivation. (D) Voltage dependence of the deactivation currents decay. (E) Voltage dependence of time to peak. (F) Voltage dependence of inactivation time constant. No significant differences were observed between control and V_H_H DI-D treated cells. hNa_v_1.1 currents: (G) I-V relationship in control (CTRL, full triangle; n = 26) and in presence of V_H_H DI-D (empty triangle; n = 42). (H) Activation and fast inactivation traces. (I) Steady state slow inactivation. (J) Voltage dependence of the deactivation currents decay. (K) Voltage dependence of time to peak. (L) Voltage dependence of inactivation time constant. No significant differences were observed between control and V_H_H DI-D treated cells. hNa_v_1.2 currents: (M) I-V relationship in control (CTRL, full diamond; n = 71) and in presence of V_H_H DI-D (empty diamond; n = 67). (N) Activation and fast inactivation traces. (O) Steady state slow inactivation. (P) Voltage dependence of the deactivation currents decay. (Q) Voltage dependence of time to peak. (R) Voltage dependence of inactivation time constant. No significant differences were observed between control and V_H_H DI-D treated cells. Data shown as mean ± SEM.

**
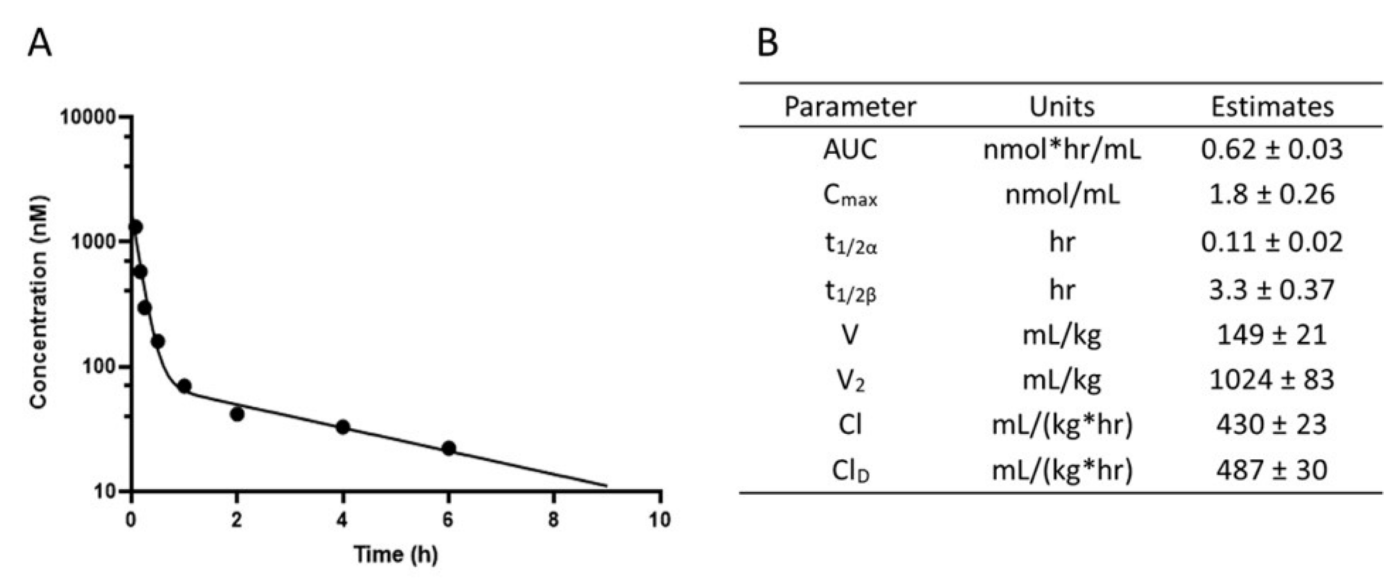
**

**Figure S10**. PK analysis of V_H_H DI-D following an intravenous bolus administration (4 mg kg^-1^) to Wistar rats. (A) Serum DI-D concentration-time profile (Mean± SEM; n = 3); Observed values (●) *vs* predicted values (-); (B) Mean PK parameter estimates (± SD) from 2-compartment analysis.

**Table S1.** Amino-acid sequences of 6 V_H_Hs Isolated.

| V_H_H | Sequence |
| --- | --- |
| DI-A | QAQVQLVESGGGLVQPGGSLRLSCAASGRTISSFTMGWFRQAPGAEREFVAAISRSGSSTVYAGSVKGRFTISRDNAKNTVYLQMNSLKPEDTAVYYCNAVITEPLYKTDDSYWGQGTQVTVSS |
| DI-B | QVKLEESGGGLVPPGESLRLTCAATGQTASVYEMAWFRRAPEKEQVYVASINWRDGDTQYHNSVKGRFIISRDNAKNTVFLQMNSLTPEDTAIYYCAARKELAGYDYWGQGTQVTVSS |
| DI-C | QVKLEESGGGLVQPGESLRLSCVGSGFNFRIQAMAWFRQAPGKEREFVASISGSGATTDHADSVKGRFAISKDNARDTMYLQMNNLKPEDTAVYYCYAISQHVPPYHYWGQGTQVTVSS |
| DI-D | QVKLEESGGGLVQPGGSLKLSCVASGFAFSSAPMDWVRKAPGKDVEWLSTIESDQDHTIYYANSVKGRFTISRDDVQNILYLQMNDLKIEDTATYYCQKRGEKKTRGQGTQVTVSS |
| DI-E | MAQVKLEESGGGLVQPGGSLRLSCAASEFTFSSSWGHWVRQAPGKGLKWVSSINSGGEGTYYADSVKGRFTISRDNGKNTLYLEMNSLKSEDTAVYYCTSASGAWGQGIQVTVSS |
| DI-H | QVKLEESGGGLVQAGESLRLSCVNSGSTFSIYAMGWYRQAPGKQRELVAAISSVGRTNYADSVKGRFTISRAGAKNTVYLHMNNLKPEDTAVYSCITYYQNAMYFGQGTQVTVSS |

The CDR3 regions are underlined.

**Table S2.** Summary of values for the effect of V_H_H DI-D on the kinetics and voltage dependence properties of the hNa_v_1.7 channels.

| Electrophysiology Parameter | Control | DI-D |
| --- | --- | --- |
| Current Amplitude at -20 mV (pA) | 2119 ± 63; *n* = 278 | 2264 ± 70; *n* = 268 |
| Activation V_1/2_ (mV) | -31.4 ± 1.8; *n* = 278 | -32.7 ± 2.0; *n* = 268 |
| Fast Inactivation V_1/2_ (mV) | -77.6 ± 0.7; *n* = 278 | -78.6 ± 0.6; *n* = 268 |
| Slow Inactivation V_1/2_ (mV) | -16.4 ± 1.6; *n* = 278 | -17.9 ±0.7; *n* = 268 |
| Deactivation Time Constant at -50 mV (ms) | 0.82 ±0.03; *n* = 202 | 0.94 ± 0.03*****; *n* = 242 |

***** Indicate a significant difference between control and V_H_H DI-D, *p* = 0.004.

**Table S3.** Summary of SPR-derived V_H_H DI-D affinities for human, mouse and rat FC5-DIE3IR.

| Human FC5-DIE3IR | | | Mouse FC5-DIE3IR | | | Rat FC5-DIE3IR | | | FC5 | | |
| --- | --- | --- | --- | --- | --- | --- | --- | --- | --- | --- | --- |
| *R*_max_* (RU) | **Chi² (RU²)** | ***K*_D_ (µm)** | ***R*_max_* (RU)** | **Chi² (RU²)** | ***K*_D_ (µm)** | ***R*_max_* (RU)** | **Chi² (RU²)** | **K_D_ (µm)** | ***R*_max_ (RU)** | **Chi² (RU²)** | ***K*_D_ (µm)** |
| 192 | 0.64 | 1.70 | 191 | 0.68 | 1.66 | 209 | 0.67 | 1.56 | - | - | n.b. |
| 192 | 0.49 | 1.72 | 191 | 0.44 | 1.70 | 209 | 0.50 | 1.59 | - | - | n.b. |
| 192 | 0.50 | 1.71 | 191 | 0.54 | 1.68 | 209 | 0.51 | 1.56 | - | - | n.b. |

*Observed *R*_max_ values. The theoretical *R*_max_ was 309, 290 and 294 RU, for human, mouse and rat, respectively. n.b.: no observed binding at up to 5 µm.

**Table S4.** Effect of V_H_H DI-D, A20.1 and TC-N 1752 in the Hargreaves model of hyperalgesia expressed as percentage of Maximal Possible Effect (%MPE).

| Percentage of Maximal Possible Effect (%MPE) | | | | | | |
| --- | --- | --- | --- | --- | --- | --- |
| Time (h) | **V_H_H A20.1** | | **V_H_H DI-D** | | **TC-N 1752** | |
|  | **50 µg (n=4)** | **100 µg (n=4)** | **50 µg (n=9)** | **100 µg (n=11)** | | **100 µg (n=8)** |
| 0 | 0.00±0.00 | 0.00±0.00 | 0.00±0.00 | 0.00±0.00 | 0.00±0.00 | |
| 1 | 4.60±3.01 | 8.71±2.54 | 29.37±4.40 | 45.51±5.38 * | 45.35±10.60 * | |
| 2 | 7.16±3.30 | 11.55±1.73 | 27.39±4.42 | 41.99±4.17 * | 47.12±8.55 * | |
| 4 | 8.24±5.64 | 10.02±3.70 | 24.38±5.42 | 45.13±3.14 *^, $^ | 44.33±4.21 * | |

* *p* < 0.05 *vs* V_H_H A20.1 (100 µg)

^$^ *p* < 0.05 *vs* V_H_H DI-D (50 µg)

**Table S5.** Summary of differential HDX kinetics

| **Amino acid numbering^1^** | | | |  |  |  |  | **1 min** | | | | | | | **10 min** | | | | | | | **60 min** | | | | | | |
| --- | --- | --- | --- | --- | --- | --- | --- | --- | --- | --- | --- | --- | --- | --- | --- | --- | --- | --- | --- | --- | --- | --- | --- | --- | --- | --- | --- | --- |
| **FC5-DIE3IR** | | **Nav1.7** | | **Peptide properties** | | | | **Control** | | **DI-D** | | **Differential HDX** | | | **Control** | | **DI-D** | | **Differential HDX** | | | **Control** | | **DI-D** | | **Differential HDX** | | |
| **Start** | **End** | **Start** | **End** | **Sequence** | **Charge** | **Mass (Da)** | **Retention  time (min)** | **Uptake  (D)** | **Uptake  SD (D)** | **Uptake  (D)** | **Uptake  SD (D)** | **Δ D Uptake^2^** | **Δ D  (%)^3^** | **1-p** | **Uptake  (D)** | **Uptake  SD (D)** | **Uptake  (D)** | **Uptake  SD (D)** | **Δ D Uptake^2^** | **Δ D  (%)^3^** | **1-p** | **Uptake  (D)** | **Uptake  SD (D)** | **Uptake  (D)** | **Uptake  SD (D)** | **Δ D Uptake^2^** | **Δ D  (%)^3^** | **1-p** |
| 21 | 36 | N/A | | SCAASGFKITHYTMGW | 3 | 1758.99 | 9.15 | 1.95 | 0.16 | 1.87 | 0.03 | -0.08 | -1.26 | 0.515 | 2.37 | 0.11 | 2.26 | 0.03 | -0.1 | -1.65 | 0.773 | 2.53 | 0.03 | 2.43 | 0.09 | -0.1 | -1.52 | 0.877 |
| 23 | 36 |  |  | AASGFKITHYTMGW | 2 | 1568.99 | 9 | 1.59 | 0.13 | 1.61 | 0.08 | 0.02 | 0.43 | 0.194 | 1.92 | 0.05 | 1.83 | 0.07 | -0.09 | -1.65 | 0.85 | 2 | 0.06 | 1.89 | 0.07 | -0.11 | -2.07 | 0.926 |
| 23 | 36 |  |  | AASGFKITHYTMGW | 3 | 1569 | 9 | 1.56 | 0.08 | 1.51 | 0.04 | -0.04 | -0.78 | 0.53 | 1.92 | 0.08 | 1.72 | 0.01 | -0.19 | -3.54 | 0.943 | 1.97 | 0.02 | 1.86 | 0.09 | -0.11 | -2.09 | 0.924 |
| 24 | 35 |  |  | ASGFKITHYTMG | 2 | 1311.83 | 7.58 | 1.41 | 0.11 | 1.48 | 0.02 | 0.06 | 1.41 | 0.564 | 1.76 | 0.04 | 1.68 | 0.02 | -0.08 | -1.76 | 0.928 | 1.85 | 0.03 | 1.77 | 0.08 | -0.08 | -1.81 | 0.853 |
| 27 | 36 |  |  | FKITHYTMGW | 2 | 1282.74 | 8.88 | 0.8 | 0.07 | 0.88 | 0.02 | 0.08 | 2.11 | 0.821 | 1.14 | 0.02 | 1.07 | 0.03 | -0.06 | -1.8 | 0.938 | 1.23 | 0.02 | 1.19 | 0.05 | -0.04 | -1.13 | 0.789 |
| 27 | 36 |  |  | FKITHYTMGW | 3 | 1282.75 | 8.88 | 0.82 | 0.05 | 0.87 | 0.03 | 0.05 | 1.44 | 0.769 | 1.1 | 0.06 | 1.08 | 0.05 | -0.02 | -0.5 | 0.282 | 1.19 | 0.02 | 1.18 | 0.06 | -0.02 | -0.47 | 0.378 |
| 28 | 36 |  |  | KITHYTMGW | 2 | 1135.67 | 8.13 | 0.54 | 0.06 | 0.61 | 0.02 | 0.07 | 2.16 | 0.828 | 0.74 | 0.03 | 0.79 | 0.04 | 0.05 | 1.48 | 0.809 | 0.86 | 0.01 | 0.87 | 0.03 | 0 | 0.02 | 0.03 |
| 28 | 36 |  |  | KITHYTMGW | 3 | 1135.68 | 8.13 | 0.45 | 0.05 | 0.48 | 0.01 | 0.03 | 0.93 | 0.614 | 0.66 | 0.01 | 0.66 | 0.02 | 0 | 0.04 | 0.066 | 0.77 | 0.01 | 0.73 | 0.04 | -0.03 | -1.01 | 0.792 |
| 36 | 46 |  |  | WFRQAPGKERE | 3 | 1402.93 | 3.56 | 1.14 | 0.1 | 1.28 | 0.03 | 0.14 | 3.98 | 0.889 | 1.45 | 0.05 | 1.35 | 0.02 | -0.11 | -2.93 | 0.951 | 1.47 | 0.04 | 1.51 | 0.03 | 0.04 | 1.13 | 0.788 |
| 37 | 46 |  |  | FRQAPGKERE | 2 | 1216.78 | 1.73 | 1.14 | 0.08 | 1.26 | 0.03 | 0.12 | 3.91 | 0.905 | 1.44 | 0.01 | 1.33 | 0.03 | -0.11 | -3.42 | 0.976 | 1.46 | 0.01 | 1.5 | 0.07 | 0.04 | 1.17 | 0.614 |
| 37 | 46 |  |  | FRQAPGKERE | 3 | 1216.82 | 1.75 | 1.15 | 0.06 | 1.29 | 0.02 | 0.14 | 4.57 | 0.964 | 1.47 | 0.04 | 1.38 | 0.01 | -0.08 | -2.69 | 0.943 | 1.5 | 0.02 | 1.54 | 0.09 | 0.04 | 1.2 | 0.543 |
| 37 | 47 |  |  | FRQAPGKEREF | 3 | 1363.84 | 3.36 | 1.33 | 0.11 | 1.43 | 0.06 | 0.1 | 2.76 | 0.755 | 1.63 | 0.03 | 1.51 | 0.02 | -0.12 | -3.41 | 0.993 | 1.67 | 0.04 | 1.7 | 0.09 | 0.04 | 1.02 | 0.48 |
| 48 | 59 |  |  | VSRITWGGDNTF | 2 | 1351.83 | 8.53 | 1.56 | 0.1 | 1.44 | 0.03 | -0.13 | -2.82 | 0.864 | 1.8 | 0.02 | 1.77 | 0.07 | -0.03 | -0.65 | 0.431 | 2.01 | 0.02 | 1.9 | 0.12 | -0.1 | -2.31 | 0.826 |
| 60 | 68 |  |  | YSNSVKGRF | 2 | 1056.7 | 3.69 | 1.27 | 0.1 | 1.38 | 0.01 | 0.12 | 3.68 | 0.812 | 1.57 | 0.04 | 1.53 | 0.08 | -0.05 | -1.52 | 0.59 | 1.67 | 0.01 | 1.69 | 0.07 | 0.02 | 0.52 | 0.306 |
| 60 | 68 |  |  | YSNSVKGRF | 3 | 1056.66 | 3.69 | 1.26 | 0.12 | 1.35 | 0 | 0.09 | 2.99 | 0.701 | 1.58 | 0.06 | 1.53 | 0.06 | -0.05 | -1.49 | 0.6 | 1.67 | 0.04 | 1.7 | 0.09 | 0.04 | 1.12 | 0.464 |
| 60 | 80 |  |  | YSNSVKGRFTISRDNAKNTVY | 3 | 2419.44 | 7.09 | 2.35 | 0.18 | 2.49 | 0.03 | 0.15 | 1.7 | 0.697 | 2.88 | 0.07 | 2.74 | 0.05 | -0.14 | -1.62 | 0.945 | 3.03 | 0.03 | 3.01 | 0.13 | -0.02 | -0.21 | 0.195 |
| 60 | 80 |  |  | YSNSVKGRFTISRDNAKNTVY | 4 | 2419.13 | 7.13 | 2.38 | 0.19 | 2.51 | 0.04 | 0.13 | 1.51 | 0.649 | 2.87 | 0.05 | 2.69 | 0.02 | -0.18 | -2.09 | 0.987 | 3.01 | 0.01 | 3.02 | 0.14 | 0.01 | 0.12 | 0.107 |
| 69 | 80 |  |  | TISRDNAKNTVY | 2 | 1380.92 | 2.72 | 1.17 | 0.07 | 1.12 | 0.02 | -0.05 | -1.03 | 0.62 | 1.28 | 0.03 | 1.2 | 0.03 | -0.08 | -1.88 | 0.967 | 1.32 | 0.01 | 1.32 | 0.06 | 0 | -0.02 | 0.02 |
| 69 | 80 |  |  | TISRDNAKNTVY | 3 | 1380.86 | 2.73 | 1.21 | 0.09 | 1.18 | 0.03 | -0.03 | -0.63 | 0.341 | 1.3 | 0.03 | 1.22 | 0.01 | -0.09 | -1.93 | 0.962 | 1.35 | 0.01 | 1.37 | 0.06 | 0.02 | 0.34 | 0.339 |
| 81 | 93 |  |  | LQMNSLKPEDTAD | 2 | 1460.81 | 6.79 | 0.55 | 0.05 | 0.46 | 0.01 | -0.09 | -2.05 | 0.92 | 0.68 | 0.04 | 0.56 | 0.02 | -0.12 | -2.56 | 0.976 | 0.81 | 0.01 | 0.73 | 0.08 | -0.08 | -1.71 | 0.862 |
| 84 | 93 |  |  | NSLKPEDTAD | 2 | 1088.65 | 2.06 | 0.35 | 0.01 | 0.34 | 0 | -0.01 | -0.24 | 0.581 | 0.38 | 0.02 | 0.33 | 0.03 | -0.04 | -1.4 | 0.877 | 0.43 | 0 | 0.41 | 0.04 | -0.03 | -0.82 | 0.705 |
| 94 (102) | 116 | (170) | 286 | YYCAAGST**K**HKCFRNSLENNETL | 4 | 2648.58 | 7.41 | 2.51 | 0.14 | 2.28 | 0.02 | -0.23 | -2.45 | 0.896 | 2.59 | 0.11 | 2.37 | 0.08 | -0.22 | -2.38 | 0.946 | 2.41 | 0.48 | 2.61 | 0.09 | 0.2 | 2.13 | 0.453 |
| 121 | 128 | 291 | 298 | NTLESEED | 1 | 935.48 | 2.36 | 1.33 | 0.07 | 1.21 | 0.01 | -0.12 | -4.45 | 0.909 | 1.33 | 0.04 | 1.22 | 0.05 | -0.11 | -4 | 0.961 | 1.27 | 0.01 | 1.24 | 0.07 | -0.03 | -1.11 | 0.532 |
| 121 | 129 | 291 | 299 | NTLESEEDF | 1 | 1082.58 | 7.71 | 1.52 | 0.08 | 1.36 | 0.03 | -0.16 | -5.17 | 0.943 | 1.52 | 0.02 | 1.37 | 0.05 | -0.14 | -4.59 | 0.977 | 1.48 | 0.02 | 1.4 | 0.06 | -0.09 | -2.71 | 0.944 |
| 121 | 129 | 291 | 299 | NTLESEEDF | 2 | 1082.58 | 7.71 | 1.47 | 0.1 | 1.02 | 0 | -0.45 | -14.27 | 0.984 | 1.44 | 0.03 | 1.05 | 0.02 | -0.38 | -12.11 | 1 | 1.44 | 0.05 | 1.06 | 0.03 | -0.37 | -11.84 | 0.999 |
| 129 | 133 | 299 | 303 | FRKYF | 2 | 759.53 | 6.97 | 0.86 | 0.02 | 0.88 | 0.02 | 0.01 | 0.95 | 0.523 | 0.94 | 0.01 | 0.88 | 0.04 | -0.06 | -4.28 | 0.902 | 0.9 | 0.02 | 0.9 | 0.03 | -0.01 | -0.54 | 0.286 |
| 129 | 134 | 299 | 304 | FRKYFY | 2 | 922.62 | 7.51 | 1.04 | 0.08 | 1.08 | 0.01 | 0.03 | 1.87 | 0.458 | 1.16 | 0.02 | 1.08 | 0.02 | -0.08 | -4.51 | 0.991 | 1.12 | 0.01 | 1.12 | 0.04 | 0 | 0.19 | 0.118 |
| 129 | 136 | 299 | 306 | FRKYFYYL | 2 | 1198.77 | 9.2 | 1.52 | 0.08 | 1.38 | 0.02 | -0.14 | -5.35 | 0.917 | 1.56 | 0.05 | 1.39 | 0.04 | -0.17 | -6.21 | 0.984 | 1.5 | 0.05 | 1.45 | 0.06 | -0.05 | -1.98 | 0.746 |
| 129 | 144 | 299 | 314 | FRKYFYYLEGSKDALL | 2 | 2012.34 | 9.28 | 3.32 | 0.18 | 3.13 | 0.03 | -0.19 | -3.04 | 0.792 | 3.5 | 0.05 | 3.16 | 0.05 | -0.34 | -5.42 | 0.999 | 3.34 | 0.03 | 3.26 | 0.1 | -0.07 | -1.13 | 0.739 |
| 129 | 144 | 299 | 314 | FRKYFYYLEGSKDALL | 3 | 2012.27 | 9.28 | 3.35 | 0.21 | 3.12 | 0.05 | -0.23 | -3.63 | 0.803 | 3.46 | 0.07 | 3.16 | 0.11 | -0.3 | -4.77 | 0.974 | 3.32 | 0.05 | 3.25 | 0.12 | -0.08 | -1.21 | 0.691 |
| 129 | 144 | 299 | 314 | FRKYFYYLEGSKDALL | 4 | 2012.27 | 9.28 | 3.1 | 0.22 | 2.88 | 0.04 | -0.22 | -3.57 | 0.787 | 3.22 | 0.07 | 2.95 | 0.08 | -0.27 | -4.22 | 0.985 | 3.11 | 0.02 | 2.97 | 0.09 | -0.13 | -2.13 | 0.951 |
| 130 | 134 | 300 | 304 | RKYFY | 2 | 775.48 | 6.18 | 0.85 | 0.02 | 0.82 | 0.02 | -0.02 | -1.82 | 0.817 | 0.91 | 0.04 | 0.84 | 0.05 | -0.07 | -5.46 | 0.864 | 0.9 | 0.01 | 0.86 | 0.06 | -0.04 | -2.62 | 0.667 |
| 134 | 143 | 304 | 313 | YYLEGSKDAL | 2 | 1157.75 | 7.69 | 1.77 | 0.08 | 1.62 | 0.02 | -0.14 | -3.99 | 0.925 | 1.77 | 0.04 | 1.56 | 0.04 | -0.21 | -5.79 | 0.997 | 1.73 | 0.02 | 1.65 | 0.05 | -0.08 | -2.3 | 0.951 |
| 135 | 144 | 305 | 314 | YLEGSKDALL | 2 | 1107.72 | 7.74 | 1.59 | 0.09 | 1.48 | 0.02 | -0.11 | -3.12 | 0.848 | 1.65 | 0.04 | 1.49 | 0.03 | -0.16 | -4.56 | 0.995 | 1.61 | 0.03 | 1.53 | 0.06 | -0.08 | -2.14 | 0.913 |
| 145 | 160 | 315 | 330 | CGFSTDSGQCPEGYTC | 2 | 1653.81 | 7.83 | 2.46 | 0.15 | 2.31 | 0.05 | -0.15 | -2.58 | 0.781 | 2.71 | 0.06 | 2.38 | 0.06 | -0.34 | -5.76 | 0.998 | 2.64 | 0.02 | 2.5 | 0.11 | -0.14 | -2.45 | 0.926 |
| 148 | 160 | 318 | 330 | STDSGQCPEGYTC | 2 | 1346.68 | 5.97 | 1.82 | 0.14 | 1.74 | 0.05 | -0.08 | -1.85 | 0.573 | 2.04 | 0.03 | 1.85 | 0.05 | -0.18 | -4.04 | 0.992 | 1.95 | 0.04 | 1.96 | 0.06 | 0.01 | 0.32 | 0.282 |
| 161 | 171 | 331 | 341 | VKIGRNPDYGY | 3 | 1280.86 | 6.37 | 1.94 | 0.13 | 1.9 | 0.05 | -0.04 | -1.03 | 0.311 | 1.93 | 0.11 | 1.87 | 0.06 | -0.07 | -1.9 | 0.574 | 1.9 | 0.05 | 1.96 | 0.07 | 0.06 | 1.64 | 0.732 |
| 161 | 175 | 331 | 345 | VKIGRNPDYGYRVDY | 2 | 1814.17 | 7.36 | 2.94 | 0.14 | 2.8 | 0.05 | -0.13 | -2.5 | 0.775 | 3.06 | 0.05 | 2.87 | 0.08 | -0.19 | -3.58 | 0.971 | 2.92 | 0.04 | 2.91 | 0.12 | 0 | -0.08 | 0.049 |
| 161 | 175 | 331 | 345 | VKIGRNPDYGYRVDY | 4 | 1814.12 | 7.36 | 2.79 | 0.18 | 2.68 | 0.01 | -0.12 | -2.13 | 0.626 | 2.9 | 0.04 | 2.72 | 0.07 | -0.18 | -3.33 | 0.977 | 2.8 | 0.08 | 2.8 | 0.1 | -0.01 | -0.11 | 0.069 |
| 171 | 175 | 341 | 345 | YRVDY | 1 | 714.46 | 5.72 | 0.69 | 0.03 | 0.75 | 0 | 0.06 | 4.4 | 0.917 | 0.82 | 0.02 | 0.79 | 0.05 | -0.03 | -2.58 | 0.622 | 0.79 | 0.03 | 0.79 | 0.03 | 0 | 0.1 | 0.043 |
| 171 | 175 | 341 | 345 | YRVDY | 2 | 714.45 | 5.73 | 0.68 | 0.04 | 0.76 | 0.03 | 0.08 | 5.74 | 0.943 | 0.82 | 0.02 | 0.8 | 0.04 | -0.02 | -1.26 | 0.409 | 0.81 | 0.02 | 0.8 | 0.04 | -0.01 | -0.89 | 0.342 |

Significant increases and decreases in deuteration upon binding are shown in red and blue, respectively.

^1^ Peptide numbering in the context of both the grafted FC5-DIE3IR construct and wild-type human Na_v_­1.7 sequence. Parentheses indicate the starting residue of the grafted DIE3IR within a peptide.

^2^ Differential deuterium uptake is calculated using the following formula: $\Delta D= D_{DI-D}- D_{Control}$

^3^ Uptake is normalized to the maximum possible deuterium content (45%) of each peptide.

**Table S6**. Summary of BLAST search results for Query sequence DIE3IR (70 aa) and NTLESEEDFRKYFY.

| Query Sequence: KHKCFRNSLENNETLESIMNTLESEEDFRKYFYYLEGSKDALLCGFSTDSGQCPEGYTCVKIGRNPDYGY | | | | | | | | | |
| --- | --- | --- | --- | --- | --- | --- | --- | --- | --- |
| Sequence ID | **Protein** | **aa** | **Sequence Alignment** | **aa** | **Score** | **Expect** | **Identities** | **Positives** | **Gaps** |
|  | Query | 1 | KHKCFRNSLENNETLESIMNTLESEEDFRKYFYYLEGSKDALLCGFSTDSGQCPEGYTCVKIGRNPDYGY | 70 | 152 bits (384) | 1.00E-45 | 70/70 (100%) | 70/70 (100%) | 0/70 (0%) |
| KAI2525611.1 | **hNav1.7** | 272 | KHKCFRNSLENNETLESIMNTLESEEDFRKYFYYLEGSKDALLCGFSTDSGQCPEGYTCVKIGRNPDYGY | 341 |  |  |  |  |  |
|  | Difference |  | KHKCFRNSLENNETLESIMNTLESEEDFRKYFYYLEGSKDALLCGFSTDSGQCPEGYTCVKIGRNPDYGY |  |  |  |  |  |  |
|  | Query | 5 | FRNSLENNETL - - - - ESIMNTLESEEDFRKYFYYLEGSK DALLCGFSTDSGQCPEGYTCVKIGRNPDYGY | 70 | 84.7 bits (208) | 6.00E-20 | 42/70 (60%) | 49/70 (70%) | 5/70 (7%) |
| KAI2525567.1 | **hNav1.2** | 296 | FNNSLDGNGTTFNRTVSIFNWDEYIED-KSHFYFLEGQNDALLCGNSSDAGQCPEGYICVKAGRNPNYGY | 364 |  |  |  |  |  |
|  | Difference |  | F NSL+ N T SIN E ED + +FY+LEG DALLCG S+D+GQCPEGY CVK GRNP +YGY |  |  |  |  |  |  |
|  | Query | 32 | FYYLEGSKDALLCGFSTDSGQCPEGYTCVKIGRNPDYGY | 70 | 76.6 bits (187) | 4.00E-17 | 29/39 (74%) | 34/39 (87%) | 0/39 (0%) |
| NP_000325.4 | **hNav1.4** | 348 | FYFLEGSNDALLCGNSSDAGHCPEGYECIKTGRNPNYGY | 386 |  |  |  |  |  |
|  | Difference |  | FY+LEGS DALLCG S+D+G CPEGY C+K GRNP+YGY |  |  |  |  |  |  |
|  | Query | 7 | NSLENNETLE - - -SIMNTLESEEDFR- - -- - - KYFYYLEGSKDALLCGFSTDSGQCPEGYTCVKIGRNPDYGY | 70 | 76.6 bits (187) | 4.00E-17 | 37/73 (51%) | 50/73 (68%) | 9/73 (12%) |
| NP_001159435.1 | **hNav1.1** | 290 | HSIEKNITVNYNGTLINETVFEFDWKSYIQDSRYHYFLEGFLDALLCGNSSDAGQCPEGYMCVKAGRNPNYGY | 362 |  |  |  |  |  |
|  | Difference |  | +S+E N T+ +++N E D++ +Y Y+LEG DALLCG S+D+GQCPEGY CVK GRNP +YGY |  |  |  |  |  |  |
|  | Query | 5 | FRNSLENNETLESIMNTLESEEDF - - -RKYFYYLEGSKDALLCGFSTDSGQCPEGYTCVKIGRNPDYGY | 70 | 75.5 bits (184) | 1.00E-16 | 32/69 (46%) | 47/69 (68%) | 3/69 (4%) |
| AAK00219.1 | **hNav1.3** | 295 | FNGTMDSNGTFVNVTMSTFNWKDYIGDDSHFYVLDGQKDPLLCGNGSDAGQCPEGYICVKAGRNPNYGY | 363 |  |  |  |  |  |
|  | Difference |  | F + + + + N T ++ + + + D+ +FY L+G KD LLCG +D+ GQCPEGY CVK GRNP+ YGY |  |  |  |  |  |  |
| Query Sequence: NTLESEEDFRKYFY | | | | | | | | | |
|  | Query | 1 | NTLESEEDFRKYFY | 14 | 51.1 bits (113) | 5.00E-09 | 14/14 (100%) | 14/14 (100%) | 0/14 (0%) |
| KAI2525611.1 | **hNav1.7** | 334 | NTLESEEDFRKYFY | 347 |  |  |  |  |  |
|  | Difference |  | NTLESEEDFRKYFY |  |  |  |  |  |  |
|  | Query | 4 | ESEEDFRKYF | 13 | 26.1 bits (54) | 4.2 | 7/10 (70%) | 8/10 (80%) | 0/10 (0%) |
| XP_016880336.1 | **TEX2** | 47 | EEEEEFREYF | 56 |  |  |  |  |  |
|  | Difference |  | E EE+ FR YF |  |  |  |  |  |  |

Protein BLAST: search protein databases using a protein query (nih.gov). Database: non-redundant protein sequences. Organism: Homo sapiens (taxid: 9606). Algorithm: blast (protein-protein BLAST). Abbreviation: TEX2, testis-expressed protein 2 isoform X5.
